# Urban wastewater overflows as hotspots for dissemination of bacteria producing extended-spectrum β-lactamases and carbapenemases in the Suquía River, Argentina

**DOI:** 10.3389/fmicb.2025.1669531

**Published:** 2025-09-24

**Authors:** Susana Eugenia Ruiz, Fabrizzio Nicolas Morandini, María Emilia Panzetta, Flavio Gabriel Lipari, María Gabriela Irrazábal, Ricardo Toselli, Martín Der Ohannesian, Cristian Amieva, María Eugenia Valdes, Federico Javier Giraudo, María del Rosario Rollán, Valeria Amé, Claudia Sola, Héctor Alex Saka

**Affiliations:** ^1^Facultad de Ciencias de la Salud, Universidad Católica de Córdoba, Córdoba, Argentina; ^2^LACE Laboratorios, Córdoba, Argentina; ^3^Centro de Investigaciones en Bioquímica Clínica e Inmunología (CIBICI), Consejo Nacional de Investigaciones Científicas y Técnicas (CONICET), Córdoba, Argentina; ^4^Departamento de Bioquímica Clínica, Facultad de Ciencias Químicas, Universidad Nacional de Córdoba, Córdoba, Argentina; ^5^Department of Integrative Immunobiology, Duke University, Durham, NC, United States; ^6^Facultad de Ciencias Médicas, Universidad Nacional de Córdoba, Córdoba, Argentina; ^7^Facultad de Ciencias Agropecuarias, Universidad Católica de Córdoba, Córdoba, Argentina; ^8^Facultad de Ciencias Químicas, Centro de Química Aplicada (CEQUIMAP), Universidad Nacional de Córdoba, Córdoba, Argentina

**Keywords:** antimicrobial resistance, *Enterobacterales*, *Aeromonas*, extended-spectrum β-lactamases, carbapenemases, urban wastewater, environmental spread of resistance, antimicrobial resistance in rivers

## Abstract

Antimicrobial resistance (AMR) is a critical global challenge, yet the role of environmental dissemination of antibiotic-resistant bacteria remains underexplored, particularly in developing regions. This study investigated urban wastewater overflows from public streets as vectors for extended-spectrum-β-lactamase (ESBL)- and carbapenemase-producing *Enterobacterales* and *Aeromonas* in the Suquía River (Córdoba, Argentina). Sixty-two water samples were analyzed for coliform counts, antimicrobial susceptibility, and resistance genes. Horizontal gene transfer was assessed by conjugation. Sixty-five ESBL- and/or carbapenemase-producing isolates were recovered, including six carbapenemase producers subjected to whole-genome sequencing (WGS). Urban wastewater exhibited coliform levels >10^8^ MPN/100 mL, while river counts increased 2–5 logs at urban and downstream sites compared to upstream, where no resistant strains were detected. ESBL- and/or carbapenemase-producers occurred in ~70% of wastewater and river samples, mainly *Escherichia coli* harboring *bla_CTX-M_*. Carbapenemase producers carried *bla_KPC-2_* or *bla_NDM-1_* in *Enterobacter*, *Klebsiella*, *Citrobacter*, and *Aeromonas caviae*. WGS revealed extensive resistomes, virulence genes, and plasmid replicons, including IncU and IncA/C2 linked to carbapenemases. Conjugation confirmed plasmid-mediated transfer of β-lactamase genes, and genetic context analysis identified clinically recognized transposons. Notably, *Enterobacter kobei* and *Aeromonas caviae* from the river carried *bla_KPC-2_* on plasmidic contigs combining clinical and environmental elements, consistent with genetic exchange within aquatic ecosystems and transfer of clinically significant resistance determinants to species adapted for riverine survival. These findings identify urban wastewater overflows as AMR hotspots that facilitate the dissemination of multidrug-resistant bacteria and mobile resistance elements into urban and peri-urban aquatic environments, underscoring the need for integrated environmental AMR surveillance.

## 1 Introduction

Antimicrobial resistance (AMR) currently represents a critical global health challenge, severely limiting anti-infective treatment options, increasing the burden of healthcare costs and exacerbating mortality rates. Recent estimations indicate that at least 1.27 million deaths/year are attributable to infections caused by antibiotic-resistant bacteria worldwide (Antimicrobial Resistance, 2022). Projections predict over 10 million deaths/year due to AMR and a decrease of 2.0–3.5% in global GDP by 2050 (O’Neill, 2016). Compounding this challenge, the discovery of new antimicrobial agents has been limited over the past decades, highlighting the urgent need for effective interventions to counteract the harmful impacts of AMR (Silver, 2011; Roca et al., 2015).

*Enterobacterales* are Gram-negative bacteria that inhabit the intestinal tract of humans and animals and are significant causes of infection. Over recent decades, antibiotic resistance within *Enterobacterales* has increased substantially, positioning them as major contributors to global mortality linked to antibiotic resistance (Morosini, 2017; Antimicrobial Resistance, 2022). In this context, extended-spectrum-β-lactamase (ESBL)-producing and carbapenemase-producing *Enterobacterales* are classified by the World Health Organization (WHO) as critical priority pathogens for which new active antibiotics are urgently needed (WHO, 2024). Importantly, ESBLs and carbapenemases are frequently encoded by mobile genetic elements, such as plasmids, transposons and integrons, whose ability for horizontal gene transfer expands their potential for dissemination across bacterial species (Bush, 2018).

The persistent increase in AMR indicates that traditional approaches, which heavily emphasize human health, are insufficient to manage this complex problem. In this context, there is a growing consensus that AMR should be viewed as a multifaceted issue arising from intricate interactions among humans, animals, and the environment, a perspective known as the “One Health” approach (Laxminarayan et al., 2013; White and Hughes, 2019).

Much remains to be uncovered about the environmental dimension of AMR, especially in developing countries (Larsson et al., 2018). Studies indicate that many of the antibiotic resistance genes found in clinically important bacteria are linked to environmental origins, emphasizing the environment’s critical role in AMR (D'Costa et al., 2011; Forsberg et al., 2012). Anthropogenic activities seem to play an important role in this process. Intensive use and release of antimicrobials across sectors such as industry, agriculture, medicine, and veterinary medicine create conditions for selective pressure that promotes the emergence and spread of resistant microorganisms (Singer et al., 2016). A significant portion of the antibiotics administered to humans and animals are excreted in active form, mainly via urine and feces, accumulating in environmental matrices like surface water, groundwater, soil, and sediments. These sub-inhibitory concentrations in natural ecosystems may foster the selection of resistant strains within native microbial communities (Zhang et al., 2018; Mutuku et al., 2022). Furthermore, anthropogenic activities may aggravate the problem by releasing antibiotic-resistant bacteria and their genes to the environment (Czatzkowska et al., 2022; Larsson and Flach, 2022).

Pollution from wastewater spills containing antibiotic-resistant bacteria in public areas, though less studied, may be a significant factor driving environmental AMR (Manaia et al., 2016). Frequent wastewater overflows occur in public streets throughout the city of Córdoba, Argentina. Although official data are lacking, unofficial estimates suggest approximately 30 overflow events per day, totaling over ten thousand annually across multiple city locations (LaVoz, 2017). Water from these overflows is channeled through the city’s stormwater drainage system, which discharges runoff mainly into La Cañada stream (a tributary of the Suquía River) or directly into the urban stretch of the Suquía River. Within this context, our study aimed to investigate the potential role of urban wastewater overflows from public streets as vectors for critically important antibiotic-resistant bacteria and their impact on the Suquía River basin. In addition, we carried out detailed genomic analyses to elucidate the resistome, virulome, plasmid content and genetic structure of carbapenemase-harboring strains recovered from these sources, and performed conjugation experiments to directly assess their horizontal transfer potential.

## 2 Materials and methods

### 2.1 Study area and sample collection

A total of 62 water samples were collected between 2016 and 2023, comprising 34 urban wastewater samples from Córdoba city, 22 surface water samples from the Suquía River and 6 surface water samples from Ansenuza National Park. Wastewater samples exhibiting organoleptic properties of sewage overflows were collected from public streets in Córdoba, the second most populous city in Argentina (1.6 million inhabitants). The Suquía River flows west to east/northeast, traversing the city of Córdoba and continuing for approximately 200 km before discharging into the Mar de Ansenuza. Sampling sites along the Suquía River were strategically selected to include both pre-urban locations (upstream of the city) and urban sites, covering the river’s course through the city as well as the area influenced by the municipal wastewater treatment plant (WWTP). The Mar de Ansenuza, within Ansenuza National Park, is a highly saline endorheic lagoon covering up to 8,000 km^2^. It is the largest saline endorheic lagoon in South America and the fifth largest worldwide. Sampling points are detailed in Supplementary Figure S1. Information on individual samples is provided in Supplementary Table S1. Samples were collected in triplicate at ~5-min intervals. Each replicate (50–200 mL) was placed in sterile containers, refrigerated immediately, and processed within 24 h.

### 2.2 Coliform bacteria count

Total coliforms, fecal coliforms and *Escherichia coli* in water samples were enumerated according to the Standard Methods for the Examination of Water and Wastewater (APHA) using methods 9221A, 9221B, and 9221C for total coliforms; method 9221E for fecal coliforms; and method 9221F for *E. coli*. Results were expressed as the Most Probable Number per 100 mL (MPN/100 mL) (Lipps and Baxter, 2023).

### 2.3 Isolation of antibiotic-resistant bacteria

A volume of 0.2 mL of undiluted and serially diluted (10^−1^ to 10^−4^) samples from wastewater, Suquía River or Ansenuza National Park surface water was inoculated onto ChromID ESBL (bioMérieux) plates for presumptive identification of ESBL- and carbapenemase-producing *Enterobacterales* and *Aeromonas*. Plates were incubated at 35–37 °C for 24–48 h. For samples collected from the Suquía River and Mar de Ansenuza, an enrichment step was additionally performed by inoculating 1 mL of sample into 10 mL of nutrient broth supplemented with cefotaxime (1 mg/L), followed by incubation at 35–37 °C for 24 h. Subsequently, 0.2 mL of undiluted or serially diluted enrichment broth was plated onto ChromID ESBL agar and incubated at 35–37 °C for 24–48 h. Colonies presumptively identified as *Enterobacterales* or *Aeromonas* were subcultured onto CLDE or MacConkey agar and identified to species-level using VITEK 2 Compact (bioMérieux, GN-ID card Ref. 21341). Isolates that could not be conclusively identified with VITEK 2 Compact, were further characterized by matrix-assisted laser desorption/ionization time-of-flight mass spectrometry (MALDI-TOF MS, Bruker, bioMérieux), using the MALDI BioTyper 3.1 software (Bruker Daltonics), MBT IVD reference library (version 2023). A total of 65 ESBL- and/or carbapenemase-producing isolates were identified (Supplementary Table S2) and cryopreserved at −80 °C in Mueller-Hinton broth supplemented with 20% glycerol.

### 2.4 Antimicrobial susceptibility testing

Antimicrobial susceptibility of *Enterobacterales* to a variety of clinically relevant antibiotics was determined by disk diffusion following the Clinical&Laboratory Standards Institute (CLSI) M100 ED34 guidelines (CLSI, 2024). Antibiotics tested included: ampicillin (10 μg), ampicillin/sulbactam (10/10 μg), amoxicillin/clavulanic acid (20/10 μg), cefazolin (30 μg), cefixime (5 μg), ceftazidime (30 μg), cefotaxime (30 μg), piperacillin/tazobactam (100/10 μg), imipenem (10 μg), meropenem (10 μg), nitrofurantoin (300 μg), trimethoprim/sulfamethoxazole (1.25/23.75 μg), ciprofloxacin (5 μg), gentamicin (10 μg) and amikacin (30 μg). For *Aeromonas*, CLSI M45-ED3 guidelines for disk diffusion were followed (CLSI, 2016) for ceftazidime (30 μg), cefotaxime (30 μg), piperacillin/tazobactam (100/10 μg), imipenem (10 μg), meropenem (10 μg), trimethoprim/sulfamethoxazole (1.25/23.75 μg), ciprofloxacin (5 μg), gentamicin (10 μg) and amikacin (30 μg), while for nitrofurantoin (300 μg) CLSI recommendations for *Enterobacterales* were applied (CLSI, 2024). Ceftazidime/avibactam (10/4 μg) susceptibility was tested by disk diffusion in both *Enterobacterales* and *Aeromonas* according to the European Committee on Antimicrobial Susceptibility Testing (EUCAST) guidelines for *Enterobacterales* (EUCAST, 2024). Phenotypic confirmation of ESBL production was carried out by the double-disk synergy test (Drieux et al., 2008). Carbapenemase production was confirmed by the modified carbapenem-inactivation method (mCIM) and the EDTA-modified mCIM method (eCIM) following CLSI recommendations (CLSI, 2024). For carbapenemase-producing strains, the minimum inhibitory concentration (MIC) of meropenem was determined by E-test (AB Biodisk) following manufacturer’s instructions.

### 2.5 Mating assays

Mating experiments were used to assess horizontal transfer of β-lactamases for selected carbapenemase- and ESBL-producing strains, using *E. coli* ER1793 (rifampin resistant) as recipient strain. Conjugation mixtures were prepared at a donor-to-recipient ratio of 1:4 in physiological saline, with inocula adjusted to approximately a McFarland standard of 2. A 100 μL aliquot of the mixture was spot-inoculated onto pre-dried, cation-adjusted Mueller-Hinton agar plates and incubated at 37 °C for 24 h. Subsequently, bacterial growth was resuspended in 1 mL of physiological saline. Then, 100 μL of the suspension (undiluted and serially diluted 10^−1^ to 10^−4^) was streaked onto Mueller-Hinton plates supplemented with rifampin (50–200 μg/mL) and either cefotaxime (4 μg/mL) or meropenem (0.5–2 μg/mL) for selection. Ten putative transconjugant colonies were re-isolated from each mating assay, identified as *E. coli* and subjected to disk diffusion susceptibility testing, double-disk and mCIM tests (as described above). PCR assays targeting the relevant resistance genes were conducted on transconjugants as detailed below.

### 2.6 PCR detection of selected ESBL- and carbapenemase-genes

Genetic determinants for selected ESBL and carbapenemases were investigated by PCR using specific primers (detailed in Supplementary Table S3). The 16S rRNA gene was amplified to verify the presence of amplifiable DNA. Total bacterial DNA was extracted using the boiling method as previously described (Queipo-Ortuno et al., 2008). PCR reactions were performed with an initial denaturation step of 5 min at 95 °C, followed by 30 amplification cycles consisting of 1 min at 95 °C, 1 min at the primer-specific annealing temperature (Supplementary Table S3), and 1 min at 72 °C, with a final extension step (5 min, 72 °C). The PCR master mix was prepared to a final volume of 25 μL per reaction, containing 0.04 U of Taq DNA polymerase (Pegasus EA01M, PB-L), 1x reaction buffer, 2 mM MgCl₂, 0.2 mM of each dNTP, and 0.4 μM of each primer in molecular biology-grade water. Each reaction tube received 24 μL of master mix and 1.0 μL of DNA template. PCR products were resolved by electrophoresis on 1.0–1.5% agarose gels stained with SybrSafe (Invitrogen). Electrophoresis was conducted in 1x TAE buffer using a Bio-Rad electrophoresis chamber (100 V, 30–45 min). DNA ladder (MA02 100 bp, PB-L) was used as a molecular size marker. Amplicons were visualized using a UVP EC3 Imaging System. PCR amplifications were carried out on a Bio-Rad C1000 thermal cycler. *E. coli* ATCC 25922 was used as negative control for detection of antibiotic resistance genes. PCR reactions designed in this study were validated with reference strains *Klebsiella pneumoniae* ATCC BAA-1705 for *bla_KPC_*, *E. coli* M1857 (Servicio Antimicrobianos INEI-ANLIS Malbrán reference strains collection) for *bla_PER_*, *E. coli* M1890 (Servicio Antimicrobianos INEI-ANLIS Malbrán reference strains collection) for *bla_CTX-M_*.

### 2.7 Whole genome sequencing, bioinformatic analysis and molecular typing

Genomic DNA from carbapenemase-producing strains was extracted using a column-based purification kit (PURO-Bacteria SA0701, PB-L) following manufacturer’s instructions. DNA concentration was determined with a NanoDrop One spectrophotometer and DNA integrity was verified by electrophoresis on a 1% agarose gel in 1x TAE buffer. Purified DNA samples were subjected to WGS on the Illumina MiSeq platform (paired-end library, 150 pb insert size) at Novogene (United States). Raw sequence quality was assessed by FastQC (version 0.12.0; https://www.bioinformatics.babraham.ac.uk/projects/fastqc/). Low-quality reads were trimmed using Trimmomatic (default parameters, version 0.49) (Bolger et al., 2014). *De novo* genome assembly was performed with SPAdes (version 3.15.5) (Prjibelski et al., 2020). Genome assembly quality was evaluated for coverage and contamination using QUAST (version 5.22) (Mikheenko et al., 2018) and CheckM (version 1.2.3) (Parks et al., 2015) (Supplementary Table S4). Functional annotation was carried out with Bakta (version 1.8.1) (Schwengers et al., 2021). Species identity was confirmed by ribosomal multilocus sequence typing (rMLST) as described (Jolley et al., 2012) utilizing the online tool https://pubmlst.org/species-id. Clonal lineages were determined by multilocus sequence typing (MLST) (Jolley et al., 2018). Antimicrobial resistance genes were identified using AMRFinderPlus (Feldgarden et al., 2021). Virulence genes were detected with VFanalyzer (Liu et al., 2022) and protein BLAST (Camacho et al., 2009) against the Virulence Factor Database (Dong et al., 2024) with a cutoff value of ≥80% coverage and ≥60% identity and restricting reported genes to *Enterobacterales* and *Aeromonadaceae*. Mobile genetic elements were identified using ISfinder (Siguier et al., 2006). Plasmid analysis was performed with PlasmidFinder (version 2.0.1) (Carattoli et al., 2014), MOB-suite (Robertson and Nash, 2018) and Deeplasmid (Andreopoulos et al., 2022). To investigate the genetic context of carbapenemases, contigs containing carbapenemase-coding genes were extracted and used as query sequences for nucleotide BLAST searches against NCBI nr database (Sayers et al., 2025). Top hits where then retrieved to perform genome alignments, which were rendered using PyGenomeViz software (Shimoyama, 2024). Assembled genomes were deposited in NCBI database under accession numbers: JBMHEC000000000 (10Cfr), JBMHED000000000 (10Kmi), JBMHEE000000000 (31Ero), JBMHEF000000000 (34Eho), JBMHEG000000000 (1.4Eko), JBMHEH000000000 (4.5Aer).

### 2.8 Statistical analysis

Statistical analyses were performed using MedCalc 10.2.0.0 and GraphPad Prism 9.2.0. For comparisons of proportions, a two-tailed Chi-square test was applied. For medians, a two-tailed Mann–Whitney test was used. The specific method employed is indicated in the corresponding table or figure legend. Statistical significance was considered for *p* < 0.05.

## 3 Results

### 3.1 Coliform bacteria in urban wastewater overflows, Suquía River and Ansenuza National Park

**Figure 1:**
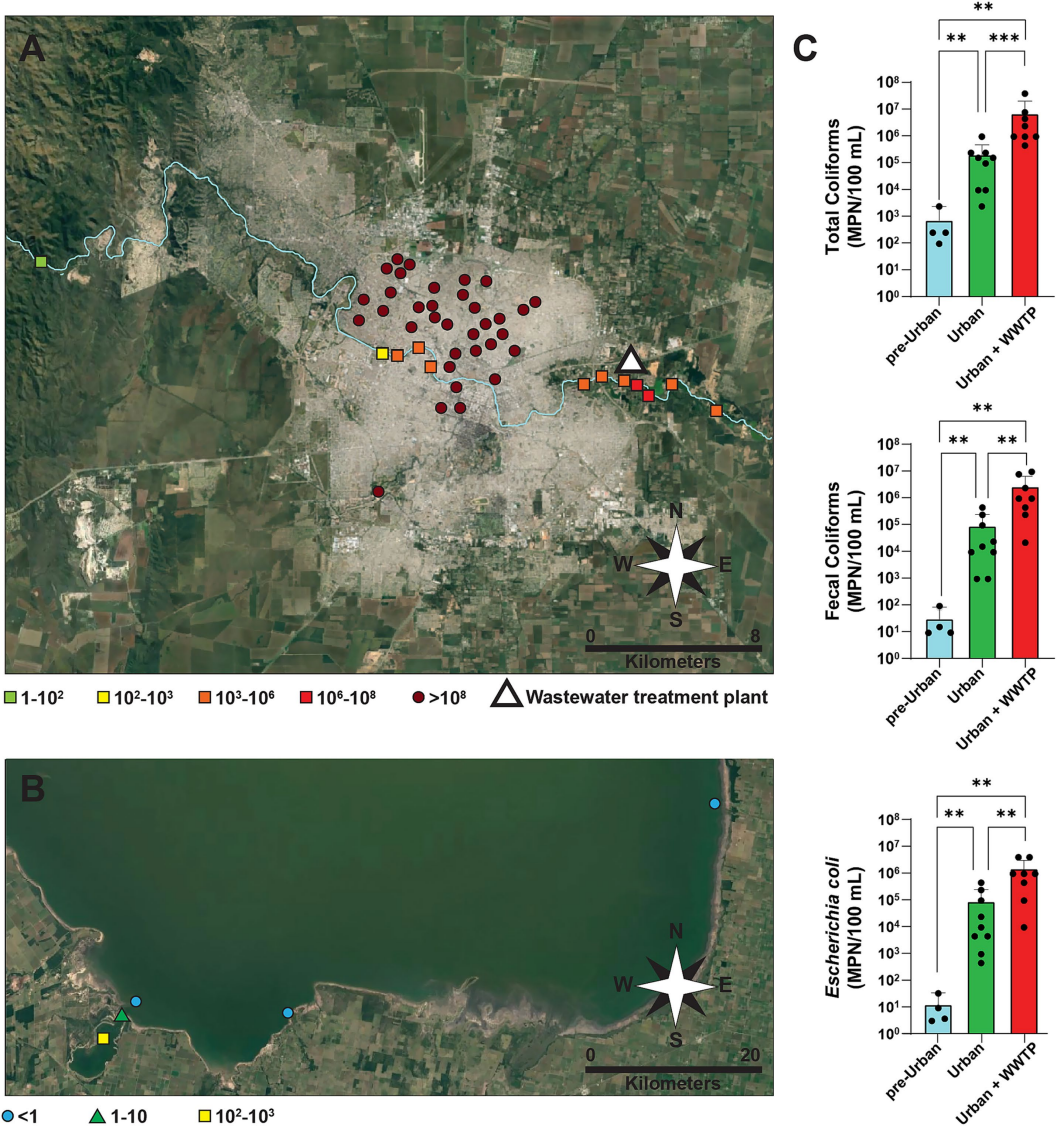
Enumeration of coliform bacteria in wastewater, surface water from the Suquía River and Ansenuza National Park sampling points. **(A)** Satellite image of Córdoba city showing wastewater overflows samples collected from public streets (circles) and surface water samples from the Suquía River (squares). The river flows from west to east. The Bajo Grande wastewater treatment plant (WWTP) is represented by a white triangle. Fecal coliform counts expressed as the most probable number per 100 mL (MPN/100 mL) are color-coded, as specified. **(B)** Satellite image of Ansenuza National Park showing surface water samples collected from the estuary of the Suquía River (Laguna del Plata) near its mouth (square), the intersection between Laguna del Plata and Mar de Ansenuza (triangle) and different locations in Ansenuza National Park (circles). Fecal coliform counts expressed as MPN/100 mL) are color-coded, as specified. **(C)** Total coliforms, fecal coliforms, and *E. coli* in surface water from the Suquía River of samples grouped into the following categories: Pre-Urban (the westernmost point, taken from the river before entering Córdoba city), Urban (seven points along the river within and exiting Córdoba city up to just before the WWTP), and Urban + WWTP (the four easternmost points, from WWTP to 10 km downstream). Columns represent the median (MPN/100 mL) and error bars correspond to the interquartile range. Black dots represent individual samples. Statistical significance was assessed using a two-tailed Mann–Whitney test, **p < 0.01, ***p < 0.001.

Frequent overflows of sewage wastewater occur in the city of Córdoba, with most discharges entering the Suquía River. As it flows through the city, the Suquía River serves as both a collector and a potential long-distance vector for wastewaters and associated microorganisms. In this context, wastewater samples were gathered from multiple points throughout the city, and surface water samples were taken from several locations along the Suquía River, covering pre-urban and urban zones before and after the impact of the city’s WWTP. To examine the potential remote effects, additional samples were collected from Ansenuza National Park, including the Suquía River estuary (Laguna del Plata) and other points within the Mar de Ansenuza lagoon (Supplementary Figure S1). To evaluate the presence of fecal bacteria, total coliforms, fecal coliforms, and *E. coli* were enumerated (Figure 1; Supplementary Table S1). All wastewater samples exhibited very high counts (>10^8^ MPN/100 mL). In contrast, samples taken from the Suquía River upstream of Córdoba city displayed fecal coliform counts ranging 10–10^2^ MPN/100 mL, several orders of magnitude lower than those found in the urban stretch of the river (10^2^–10^6^ MPN/100 mL). Further increases were detected close to the WWTP, with values staying high for at least 10 km downstream (Figure 1A; Supplementary Table S1). At the mouth of the Suquía River, the highest fecal coliform values (10^2^–10^3^ MPN/100 mL) were observed in the estuary of Laguna del Plata. These values decreased to 1–10 MPN/100 mL at the interface with the saline waters of the Mar de Ansenuza, while counts became undetectable further offshore (Figure 1B). To further investigate the impact of the city of Córdoba in the Suquía River, we performed a quantitative analysis grouping samples by location: “pre-Urban” (upstream of the city), “Urban” (within the city and up to just before the WWTP), and “Urban + WWTP” (from the WWTP to 10 km downstream). Coliform counts exhibited statistically significant increases when comparing “Urban” or “Urban + WWTP” samples to “pre-Urban” samples (Figure 1C). Significant increases were also observed between “Urban + WWTP” to “Urban” samples.

These results indicate that the city of Córdoba contributes substantial quantities of coliform bacteria to the Suquía River, with increases of 2, 3, and 4–5 orders of magnitude for total coliforms, fecal coliforms, and *E. coli*, respectively.

### 3.2 ESBL- and carbapenemase-producing bacteria are present in high proportions in urban wastewater overflows and the urban stretch of the Suquía River

**Figure 2:**
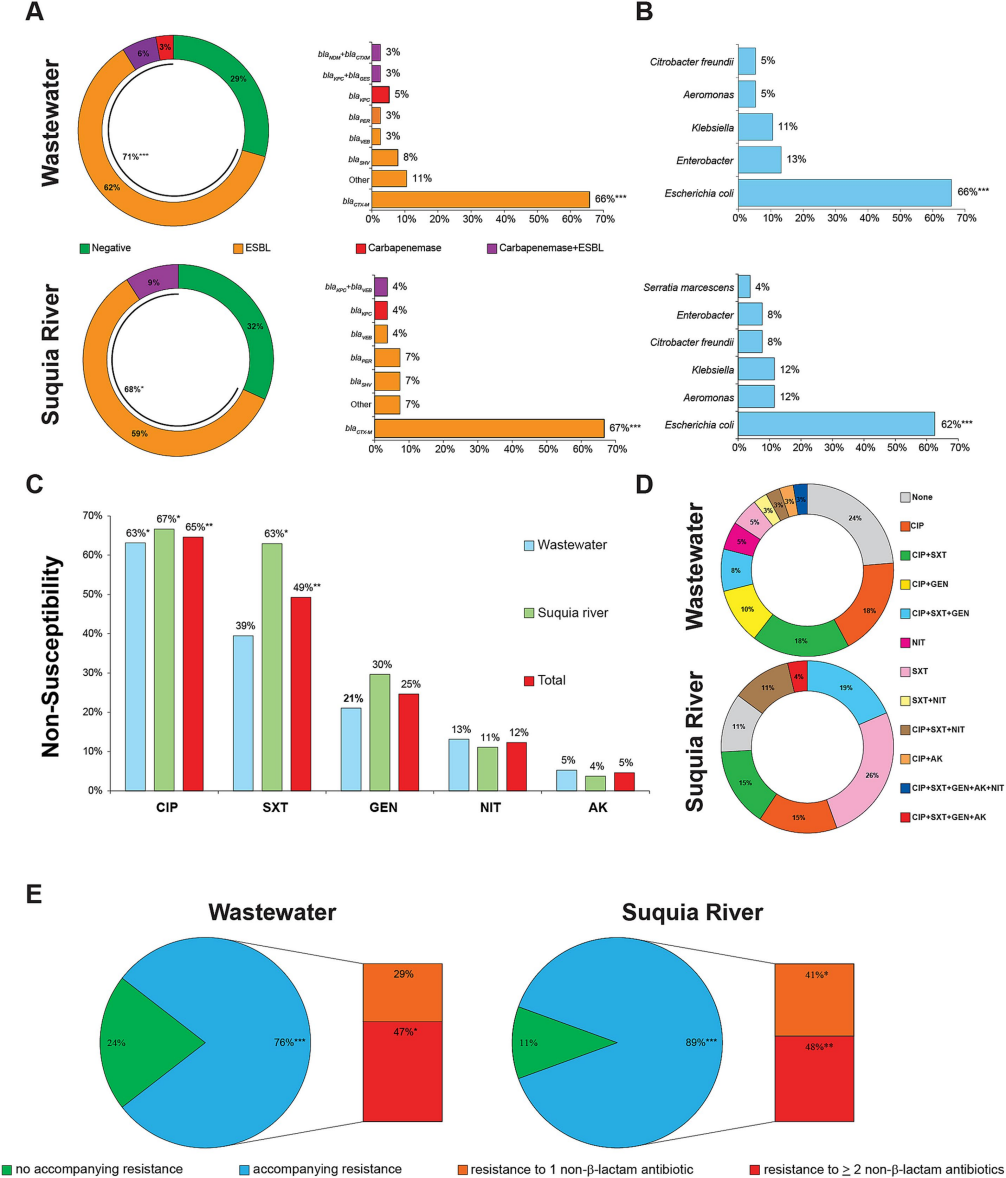
Antibiotic resistance profiles, genetic determinants and species distribution of bacteria harboring ESBLs and/or carbapenemases in wastewater overflows and surface water of the Suquía River. **(A)** Percent of wastewater overflows (*n* = 34) and Suquía River surface water (*n* = 22) samples containing ESBL and/or carbapenemase-producing bacteria and their ESBL- or carbapenemase-encoding genes. **(B)** Distribution of species harboring ESBL and/or carbapenemases in wastewater overflows (*n* = 38) and Suquía River surface water (*n* = 27). **(C)** Accompanying resistance to non-β-lactam antibiotics. CIP, ciprofloxacin; SXT, trimethoprim-sulfamethoxazole; GEN, gentamicin; NIT, nitrofurantoin; AK, amikacin. **(D,E)** Combination and percentages of accompanying resistances to non-β-lactam antibiotics, respectively. Asterisks indicate statistical significance by Chi square test (*p < 0.05, **p < 0.01, ***p < 0.001).

ESBL- and carbapenemase-producing *Enterobacterales* are classified as critical priority bacterial pathogens by the WHO (WHO, 2024), thus, their presence in water samples was investigated. Additionally, ESBL- and carbapenemase-producing *Aeromonas* spp. were studied, as these bacteria not only exhibit pathogenic potential but also, as part of aquatic microbial communities, may serve as environmental reservoirs for antibiotic resistance genes (Jones et al., 2023). A detailed list of all 65 ESBL- and/or carbapenemase-producing bacteria isolated in this study is provided in Supplementary Table S2. A high proportion of wastewater overflows collected from public streets (71%) and surface water from the Suquía River (68%) contained bacteria producing ESBL and/or carbapenemases (Figure 2A). In wastewater overflows, 62% of samples contained ESBL-producing bacteria, 6% had both ESBL- and carbapenemase-producing bacteria, and 3% contained carbapenemase producers. In surface water from the Suquía River, ESBL-producing bacteria were detected in 59% of samples, while ESBL- and carbapenemase-coproducers were identified in 9% of samples. In clear contrast, these bacteria were not detected from surface waters of the Mar de Ansenuza or from the Suquía River upstream of the city. The main genetic determinant of ESBLs was *bla_CTX-M_* (Figure 2A). Among carbapenemase-producing strains, five carried *bla_KPC_* and one harbored *bla_NDM_*. Regarding the distribution of species expressing these resistant phenotypes, ESBL-producing *E. coli* predominated (66% in wastewater and 62% in the Suquía River, respectively) (Figure 2B).

To characterize accompanying resistance not conferred by β-lactamase production, susceptibility to a variety of non-β-lactam antibiotics was determined. The two main accompanying resistances observed were to ciprofloxacin and trimethoprim-sulfamethoxazole (Figure 2C). Analysis of resistance combinations (Figure 2D) revealed 12 different profiles. Among wastewater isolates, out of 11 profiles, the top four -accounting for 54%- were: ciprofloxacin (18%), ciprofloxacin plus trimethoprim-sulfamethoxazole (18%), ciprofloxacin plus gentamicin (10%), ciprofloxacin plus trimethoprim-sulfamethoxazole plus gentamicin (8%). For Suquía River isolates, out of seven profiles, the top four -representing 75%- were: trimethoprim-sulfamethoxazole (26%), ciprofloxacin plus trimethoprim-sulfamethoxazole plus gentamicin (19%), ciprofloxacin (15%), and ciprofloxacin plus trimethoprim-sulfamethoxazole (15%). As shown in Figure 2E, 76% of wastewater and 89% of Suquía River isolates exhibited accompanying resistance. The most frequent profile was co-resistance to two or more non-β-lactam antibiotics, underscoring the multi-resistant phenotype of these strains.

KPC- and NDM-type carbapenemases confer resistance to last-resort antibiotics (Bush, 2013; Perez and Bonomo, 2019), making the isolation of environmental strains harboring these carbapenemases particularly relevant. We summarize the origin, species, MLST type, carbapenemase genes and resistance profile of the carbapenemase-producing strains isolated in the context of this study in Table 1. None of the carbapenemase-producing strains corresponded to *K. pneumoniae* -the main driver of carbapenemase dissemination in clinical settings (David et al., 2019)-, but to other *Enterobacterales* and, interestingly, *Aeromonas caviae* from the Suquía River. Notably, two of the three wastewater overflows yielding carbapenemase-producing isolates (samples AR30 and AR31, Supplementary Table S2) were collected near healthcare centers. The third one (sample AR34, Supplementary Table S2) was obtained from a large-scale sewage overflow near the urban course of the Suquía River (LaVoz, 2021). The two Suquía River surface water samples with carbapenemase-producing bacteria (RS1.4 and RS4.5, Supplementary Table S2) were collected 6 km and 10 km downstream from the WWTP. For MLST types (Table 1), the KPC-producer isolate *C. freundii* (10Cfr) was identified as the high-risk clone ST22 (Biez et al., 2023), while the rest of carbapenemase-producing *Enterobacterales* strains harbored sporadic MLST types. Interestingly, the KPC-producer *A. caviae* strain (4.5Aer) was assigned to ST2216, identified in this study. Regarding the susceptibility profiles, each strain presented a distinct resistance pattern, but all exhibited accompanying resistance to at least one non-β-lactam antibiotic. Remarkably, the NDM-producer was resistant to all 5 non-β-lactam antibiotics tested (Table 1). All KPC-producers were susceptible to ceftazidime-avibactam, as expected. Meropenem MICs ranged from 1 to >32 mg/L; the highest was observed in the NDM-producer *E. hormaechei* (34Eho), whereas KPC producers MICs ranged 1–4 mg/L.

**Table 1:** Carbapenemase-producing strains isolated from wastewater overflows (WW) and Suquía River (SR).

Strain (origin)	Species (carbapenemase)	MLST	MIC meropenem	Susceptibility to selected antibiotics					
				NIT	SXT	GEN	AK	CIP	CZA
10Cfr (WW)	*Citrobacter freundii* (*bla_KPC_*)	ST22	4 mg/L	S	R	S	S	R	S
10Kmi (WW)	*Klebsiella michiganensis* (*bla_KPC_*)	ST143	24 mg/L	S	S	I	S	I	S
31Ero (WW)	*Enterobacter roggenkampii* (*bla_KPC_*)	ST40	2 mg/L	S	S	R	R	R	S
34Eho (WW)	*Enterobacter hormaechei* (*bla_NDM_*)	ST121	>32 mg/L	R	R	R	R	R	R
1.4Eko (SR)	*Enterobacter kobei* (*bla_KPC_*)	ST691	1 mg/L	S	R	S	S	S	S
4.5Aer (SR)	*Aeromonas caviae* (*bla_KPC_*)	ST2216*	2 mg/L	S	S	S	S	R	S

Multilocus sequence types (MLST), minimum inhibitory concentration (MIC) to meropenem and susceptibility phenotypes to selected antibiotics are indicated. S, susceptible; I, intermediate; R, resistant; NIT, nitrofurantoin; SXT, trimethoprim-sulfamethoxazole; GEN, gentamicin; AK, amikacin; CIP, ciprofloxacin; CZA, ceftazidime-avibactam. *Newly identified sequence type.

These findings demonstrate that urban wastewater overflows from Córdoba, as well as surface water from the urban stretch of the Suquía River up to 10 km downstream of the city’s WWTP, carry viable ESBL- and/or carbapenemase-producing *Enterobacterales*, underscoring the environmental dissemination of critical-priority resistant bacteria.

### 3.3 β-lactamase genes are horizontally transferred by ESBL- and carbapenemase-producing environmental strains in mating assays

We performed conjugation assays using all carbapenemase-producing and one representative ESBL-producing strain to evaluate their ability to horizontally transfer β-lactamase genes (Table 2). Successful carbapenemase transfer was achieved for three strains: KPC-producers *C. freundii* (10Cfr) and *K. michiganensis* (10Kmi), and NDM-producer *E. hormaechei* (34Eho), the latter also co-transferring gentamicin and amikacin resistance. For strains co-producing KPC and ESBLs, *E. roggenkampii* (31Ero) and *E. kobei* (1.4Eko), only the ESBL determinants (*bla_GES_* and *bla_VEB_*, respectively) were transferred. No transfer was detected for the KPC-producing *A. caviae* strain despite repeated attempts. Additionally, the *bla_CTX-M_* determinant from the representative ESBL-producing strain *K. pneumoniae* (18Kpn) was successfully transferred, with co-transfer of gentamicin and trimethoprim-sulfamethoxazole resistance.

**Table 2:** Transferability of carbapenemase and ESBL determinants in mating assays.

Donor strain ID (origin)	Donor strain species (determinant/s of interest)	Donor strain accompanying resistance	Transconjugant strain ID (determinant/s of interest transferred)	Transconjugant strain accompanying resistance
10Cfr (WW)	*Citrobacter freundii* (*bla_KPC_*)	CIP, SXT	! C10Cfr (*bla_KPC_*)	None
10Kmi (WW)	*Klebsiella michiganensis* (*bla_KPC_*)	CIP, GEN	! C10Kmi (*bla_KPC_*)	None
31Ero (WW)	*Enterobacter roggenkampii* (*bla_KPC_*, *bla_GES_*)	GEN, AK	! C31Ero (*bla_GES_*)	None
34Eho (WW)	*Enterobacter hormaechei* (*bla_NDM_, bla_CTXM_*)	CIP, GEN, AK, NIT, TMS	! C34Eho (*bla_NDM_*)	AK, GEN
1.4Eko (SR)	*Enterobacter kobei* (*bla_KPC_, bla_VEB_*)	None	! C1.4Eko (*bla_VEB_*)	None
4.5Aer (SR)	*Aeromonas caviae* (*bla_KPC_*)	CIP	None obtained	Not applicable
18Kpn (WW)	*Klebsiella pneumoniae* (*bla_CTX-M_*)	CIP, GEN, SXT	! C18Kpn (*bla_CTX-M_*)	GEN, SXT

Mating assays were performed using carbapenemase- and ESBL-producing strains isolated from wastewater overflows (WW) and the Suquía River (SR) as donors. *E. coli* ER1793 was used as the recipient strain. Resistance determinants and accompanying resistance to non-β-lactam antibiotics of donor and transconjugant strains are indicated. CIP, ciprofloxacin; GEN, gentamicin; AK, amikacin, NIT, nitrofurantoin; SXT, trimethoprim-sulfamethoxazole.

These results indicate that ESBL and carbapenemase genes in *Enterobacterales* from wastewater overflows and the Suquía River are mainly encoded on transferable plasmids, highlighting their potential for horizontal dissemination.

### 3.4 Carbapenemase-producing isolates from urban wastewater and the Suquía River harbor extensive resistomes and virulomes

**Figure 3:**
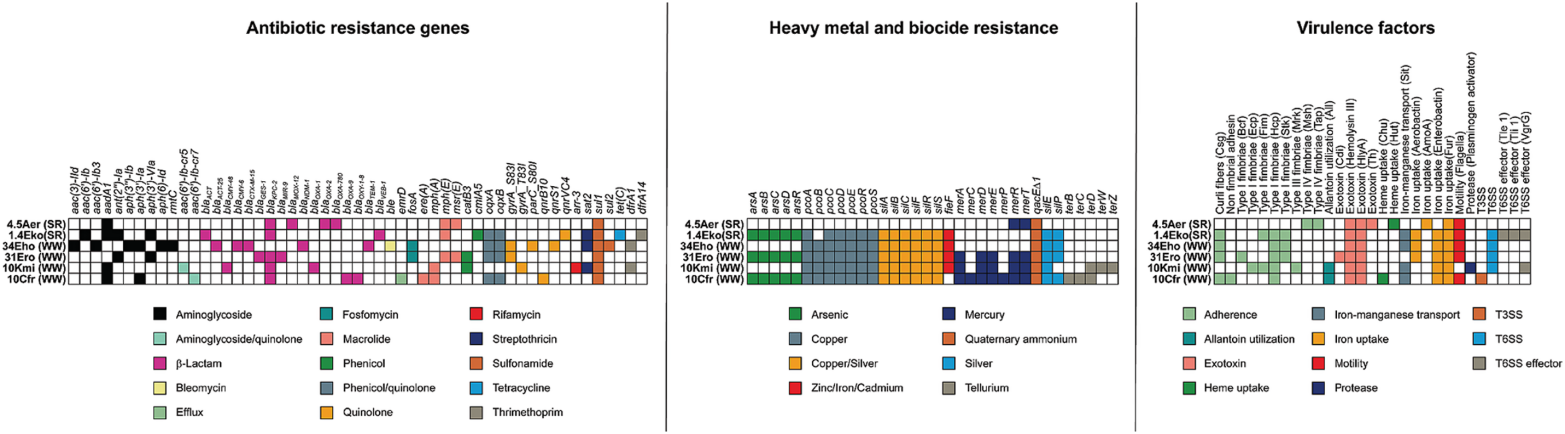
Antibiotic, heavy metals and biocide resistance genes and virulence determinants in carbapenemase-producing bacteria isolated from urban wastewater overflows (WW) and the Suquía River (SR). The presence of genes encoding antibiotic resistance (left panel), resistance to biocides and heavy metals (middle panel), and virulence factors (right panel) are indicated.

WGS analysis of all carbapenemase-producing strains (*n* = 6) revealed a broad repertoire of antibiotic resistance genes. All isolates carried genes conferring resistance to multiple antibiotics commonly used in clinical practice, consistent with their multidrug-resistant profiles (Figure 3; Supplementary Table S5). Notably, all strains harbored resistance determinants for at least four antibiotic classes, with resistance genes to β-lactams and aminoglycosides being the most numerous and diverse. Collectively, 53 genetic determinants conferring resistance to at least 12 different antibiotic classes were identified. Resistance gene counts varied among strains, ranging from 8 in KPC-producing *A. caviae* (4.5Aer) from the Suquía River to 22 in NDM-producing *E. hormaechei* (34Eho) from wastewater. Remarkably, all strains carried 3–5 β-lactamase genes. *K. michiganensis* (10Kmi) and *C. freundii* (10Cfr), recovered from a wastewater overflow near a healthcare center, carried *bla_KPC-2_*. In addition to its intrinsic β-lactamase *bla_KOXY_*, *K. michiganensis* (10Kmi) harbored the carbenicillinase *bla_OXA-9_*. Meanwhile, *C. freundii* (10Cfr) possessed its intrinsic cephalosporinase *bla_CMY-48_* and the carbenicillinase *bla_OXA-1_*. *E. roggenkampii* (31Ero), isolated from wastewater near another healthcare center, also carried the *bla_KPC-2_* carbapenemase. Alongside its natural cephalosporinase *bla_MIR-9_*, this isolate presented *bla_GES-1_*, a relatively rare ESBL. Regarding *E. hormaechei* (34Eho), recovered from wastewater overflow near the Suquía River, in addition to the *bla_NDM-1_* carbapenemase, it harbored its intrinsic cephalosporinase *bla_ACT_*, as well as three additional β-lactamases: *bla_CMY-6_*, *bla_TEM-1_* and the ESBL *bla_CTX-M-15_*. Carbapenemase-producing strains recovered from the Suquía River, *E. kobei* (1.4Eko) and *A. caviae* (4.5Aer), both carried *bla_KPC-2_* in addition to their species-specific AmpC cephalosporinases (*bla_ACT_* and *bla_MOX_*, respectively). *E. kobei* (1.4Eko) also carried *bla_VEB-1_* (an infrequent ESBL) and *A. caviae* (4.5Aer) harbored *bla_OXA-2_* and *bla_OXA-780_* (narrow-spectrum class D carbenicillinases). The detection of a *bla_KPC-2_*-producing *A. caviae* isolate in the Suquía River was particularly interesting, as this species is a natural inhabitant of aquatic microbial communities (Fernandez-Bravo and Figueras, 2020) and this isolate represents, to our knowledge, the first report of a KPC-producing *A. caviae* strain from a river in Argentina. In summary, all KPC-producing strains carried *bla_KPC-2_*, while the NDM-producing strain carried *bla_NDM-1_.* Importantly, both carbapenemases are widely disseminated in clinical settings in Argentina and globally (Hammoudi Halat and Ayoub Moubareck, 2020). Also, all strains encoded narrow-spectrum β-lactamases (*bla_TEM-1_*, *bla_OXA-1_*, *bla_OXA-2_*, *bla_OXA-9_*, and/or *bla_OXA-780_*), with three also carrying ESBLs (*bla_GES-1_*, *bla_VEB-1_*, or *bla_CTX-M-15_*). Aminoglycoside resistance profiles varied: *A. caviae* (4.5Aer) contained only *aadA1*, while others had multiple aminoglycoside resistance genes (nucleotidyl/acetyl/phosphotransferases). Notably, the NDM-producing strain *E. hormaechei* (34Eho) contained six aminoglycoside resistance determinants, including the 16S methyltransferase *rmtC*.

Biocides, heavy metal resistance and virulence genes were analyzed (Figure 3; Supplementary Table S5), since they are important for bacterial survival in clinical and environmental settings. All carbapenemase-producing isolates carried *qacEΔ1* (encoding an efflux pump for quaternary ammonium disinfectants). Except for *A. caviae* (4.5Aer), all strains also carried the *pco* and *sil* systems (copper and silver resistance, respectively). Resistance determinants for arsenic, mercury, tellurium and zinc/iron/cadmium exhibited strain-specific variability. All isolates encoded virulence factors across at least five functional categories, including adhesins, exotoxins, and iron uptake systems. The Type VI secretion system (T6SS) was found in *Enterobacter* strains (1.4Eko, 34Eho, 31Ero) and *K. michiganensis* (10Kmi), which lacked flagellar genes. Surprisingly, the KPC-producing high-risk clone *C. freundii* ST22 (10Cfr) encoded a Type III secretion system with structural genes and the effector *ipaC/sipC*.

Overall, these findings demonstrate that carbapenemase-producing strains isolated from urban wastewater overflows and Suquía River surface water harbor extensive genetic repertoires for antibiotic resistance and virulence, underscoring their multidrug-resistant profiles and pathogenic potential.

### 3.5 Carbapenemase-producing isolates encode multiple plasmid replicons

Plasmid replicon analysis revealed that all carbapenemase-producing strains harbored multiple plasmid types, ranging from 2 in *A. caviae* (4.5Aer) to 7 in *E. kobei* (1.4Eko) (Supplementary Table S6). The Col440I replicon was present in all strains except *K. michiganensis* (10Kmi), indicating its widespread distribution. The IncL/M replicon, identified in *C. freundii* (10Cfr), *K. michiganensis* (10Kmi) and *E. roggenkampii* (31Ero), represented the second most broadly distributed plasmid type. Importantly, the IncU replicon identified in *E. kobei* (1.4Eko) was located on the contig encoding *bla_KPC-2_*, while the IncA/C2 replicon in *E. hormaechei* (34Eho) was found within the contig carrying *bla_NDM-1_*, indicating the plasmid-mediated dissemination of these resistance genes.

These results demonstrate that carbapenemase-producing strains isolated from urban wastewater overflows and the Suquía River possess a variety of plasmid types, including those encoding carbapenemases, underscoring their capacity for horizontal transfer of critically important antibiotic resistance.

### 3.6 Genetic context of carbapenemase genes in environmental strains include clinically recognized arrangements

**Figure 4:**
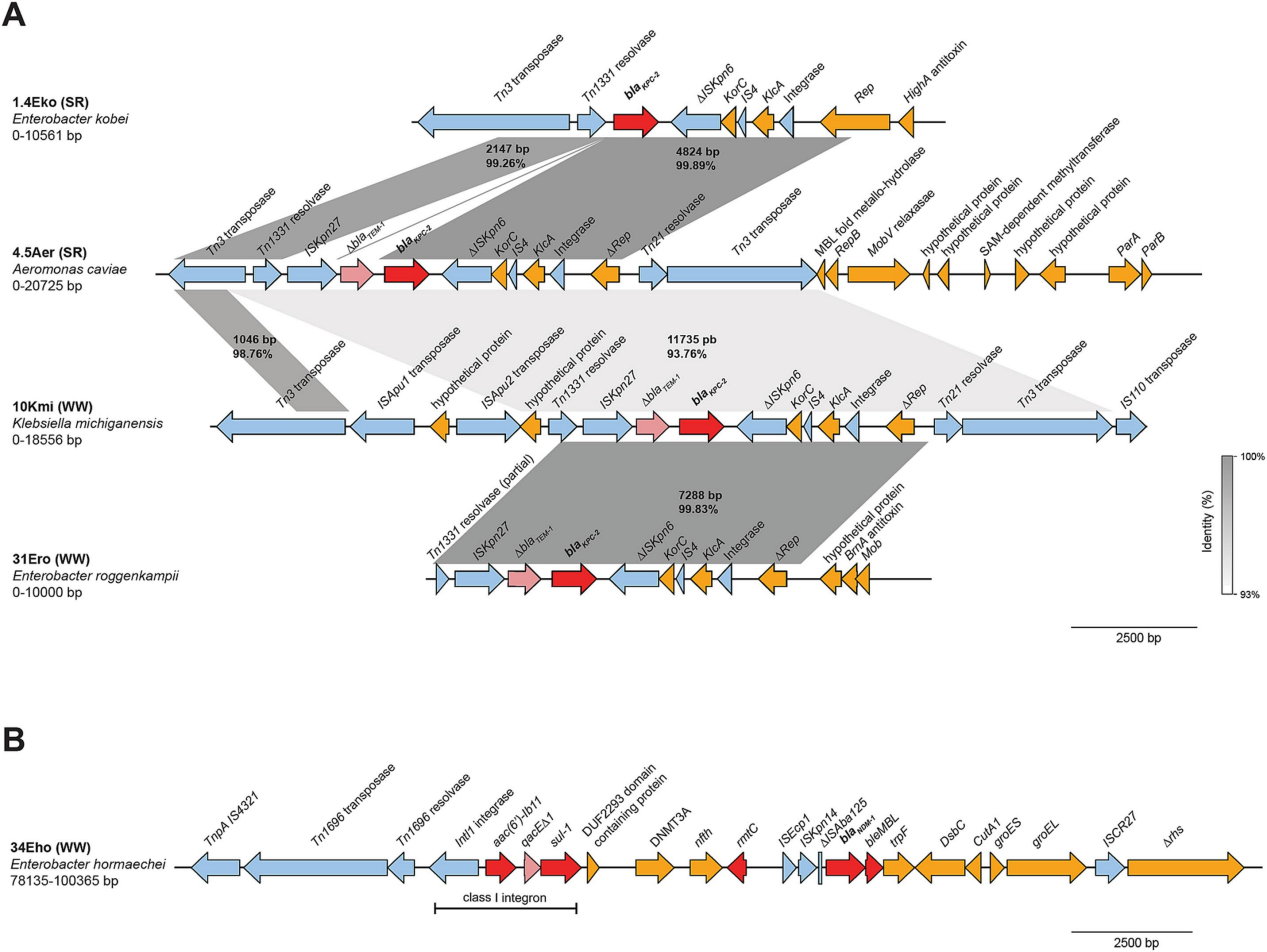
Genetic context of carbapenemases identified in strains isolated from wastewater overflows (WW) and Suquía River (SR). Linear arrangements of DNA sequences corresponding to the genetic context of *bla_KPC-2_* **(A)** and *bla_NDM-1_* **(B)** are shown. Gene annotations are depicted as colored arrows: antimicrobial resistance genes (red), truncated antimicrobial resistance genes (light red), insertion sequences, transposases, resolvases and integrases (light blue), all other genes (gold). Shaded boxes indicate the percent identities of aligned regions. Scale bars represent DNA size in base pairs (bp).

Detailed analysis of the sequences surrounding *bla_KPC-2_* in strains recovered from wastewater overflows and the Suquía River (Figure 4A), showed that *K. michiganensis* (10Kmi), *E. roggenkampii* (31Ero) and *A. caviae* (4.5Aer) carry the same transposon. This transposon is characterized by flanking insertion sequences *ISKpn27* and *ΔISKpn6*, with *Δbla_TEM-1_* upstream of *bla_KPC-2_*. This genetic arrangement, *ΔISKpn6-Δbla_TEM-1_-bla_KPC-2_-ISKpn27*, has been reported in clinical KPC-producing *Enterobacterales* in Argentina and other South American countries and is referred to as the “Non-Tn4401 variant 1a” (Gomez et al., 2011; Reyes et al., 2020). Interestingly, BLAST analysis shows that this genetic context has also been detected in an *Enterobacter asburiae* isolate from raw sewage in Argentina [strain WW-19C, CP080110.1 (Ghiglione et al., 2021)]. In contrast, *E. kobei* (1.4Eko) lacks *Δbla_TEM-1_* and *ΔISKpn27* upstream of *bla_KPC-2_*, suggesting a native variant. For *C. freundii* (10Cfr), a complete characterization of the genetic context was not possible due to insufficient sequence coverage upstream of *bla_KPC-2_*. The *bla_NDM-1_* gene in *E. hormaechei* (34Eho) resides within the ARI-A resistance island (Figure 4B), featuring a complex hybrid transposon with *ΔISAba125*, *ISKpn14*, *ISEcp1*, and a class I integron upstream, as previously described on IncC plasmids from clinical NDM-producing *Enterobacterales* in Argentina (Weber et al., 2019).

These findings indicate that carbapenemase genes in strains from wastewater overflows and the Suquía River are mobilized by transposon elements, including clinically recognized genetic arrangements.

### 3.7 Plasmid localization and genetic structure analysis of carbapenemase-harboring contigs

Since the *bla_KPC_* and *bla_NDM_* carbapenemase genes are predominantly plasmid-borne (Bush, 2018), we employed the deep learning-based bioinformatic tool Deeplasmid to predict their plasmid localization. This tool has been demonstrated to accurately distinguish plasmid sequences from bacterial chromosomal DNA (Andreopoulos et al., 2022). As shown in Supplementary Table S7, Deeplasmid analysis predicted plasmid localization with high-confidence scores (approaching 1.0) for carbapenemase-bearing contigs. *C. freundii* (10Cfr) was excluded from this analysis because its contig consisted almost entirely of the transposable element carrying *bla_KPC-2_*, precluding reliable plasmid prediction. Nevertheless, conjugation assays confirm that *bla_KPC-2_* is plasmid-encoded in this strain.

**Figure 5:**
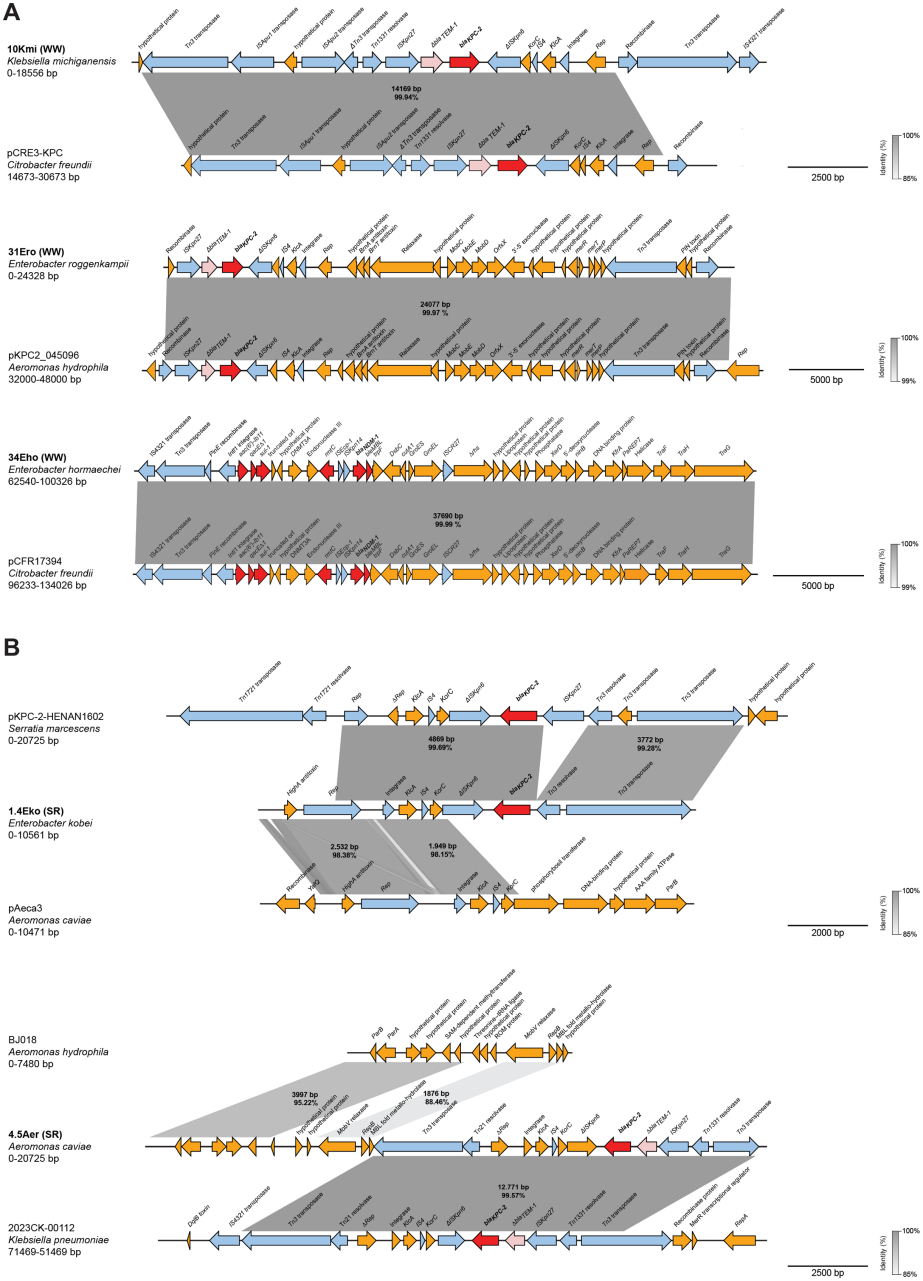
Genetic structure and comparative analysis of carbapenemase genes-harboring contigs. Linear arrangements of contigs harboring carbapenemase genes identified in strains isolated from **(A)** wastewater overflows (WW) and **(B)** Suquía River (SR) aligned with highly homologous sequences are shown. Gene annotations are depicted as colored arrows: antimicrobial resistance genes (red), truncated antimicrobial resistance genes (light red), insertion sequences, transposases, resolvases and integrases (light blue), all other genes (gold). Shaded boxes indicate the percent identities of aligned regions. Scale bars represent DNA size in base pairs (bp).

To further characterize the genetic architecture of these resistance determinants, we performed BLASTn-based comparative analyses of the carbapenemase-containing contigs. Analysis of strains from wastewater overflows (Figure 5A) showed *K. michiganensis* (10Kmi) and *E. roggenkampii* (31Ero) *bla_KPC-2_* contigs shared >99.9% identity with IncP6 plasmids pCRE-KPC (MH919378.1) (Dong et al., 2020) and pKPC2_045096 (CP028566.1), respectively, from clinical and environmental strains in China. While both 10Kmi and 31Ero strains, shared high sequence identity and coverage with IncP6 plasmids from *Aeromonas* (data not shown), this replicon was not detected in the mentioned strains. The *bla_NDM-1_* contig in *E. hormaechei* (34Eho) included multiple resistance genes and matched the IncA/C2 conjugative plasmid pCFR17394 (MH995506.1) from a clinical *C. freundii* previously isolated in Argentina (Martino et al., 2019). For Suquía River strains (Figure 5B), the top BLAST hit for *bla_KPC-2_* contig *of E. kobei* (1.4Eko) corresponded to a clinical *Serratia marcescens* plasmid pKPC-2-HENAN1602 from China (CP047392.1), with an upstream IncU replicon matching a small *A. caviae* plasmid pAeca3 (CP039626.1) lacking antibiotic resistance genes and identified from wastewater also in China. Lastly, analysis of the *bla_KPC-2_* contig of *A. caviae* (4.5Aer) revealed a 12,771 bp fragment sharing over 99% identity to an IncL/M-type plasmid from a U. S. clinical *K. pneumoniae* ST307 isolate (CP137926.1). Similarly to 1.4Eko, immediately upstream of this region, the top hit corresponded to a small *A. hydrophila* plasmid (CP180566.1) lacking resistance genes. These genetic arrangements combining clinical and environmental elements, are consistent with integration of carbapenemase genes into native plasmids via genetic exchange in the environment.

Together, these analyses indicate that carbapenemase genes in these environmental isolates reside on plasmids, including genetic structures consistent with combined clinical and environmental sequences in strains from the Suquía River.

## 4 Discussion

This study provides new insights into the environmental dissemination of AMR by characterizing ESBL- and carbapenemase-producing *Enterobacterales* and *Aeromonas* in urban wastewater overflows and Suquía River surface waters in Córdoba, Argentina. Results show that wastewater overflows release high levels of coliforms and *E. coli.* Interestingly, this observation coincides with increased coliform counts along the river’s urban stretch. Further rises were found near the city WWTP, a relatively common occurrence in low-resource settings due to overloads and/or infrastructure issues (Wang et al., 2022; Lee et al., 2023). Thus, our findings strongly suggest that wastewater overflows introduce coliform bacteria into the river, likely via the stormwater system, representing an entry point of contamination with antibiotic-resistant bacteria. Persistence of high coliform levels up to 10 km downstream of the WWTP support the idea that these bacteria can remain viable during river transport, creating an extended contamination zone. Although counts declined near the river’s mouth and approached detection limits in the hypersaline Mar de Ansenuza lagoon, the observed distribution underscores the potential for long-distance dissemination of AMR determinants through interconnected aquatic environments.

Bacteria resistant to critically important antibiotics were detected in ~70% of urban wastewater overflows and Suquía River samples. ESBL production (~60%) was most common, mainly linked to *E. coli* carrying *bla_CTX-M_*, while carbapenemases (~9%) occurred in *Enterobacter*, *Klebsiella*, *Citrobacter*, and *Aeromonas* via *bla_KPC_* or *bla_NDM_*. These findings are in line with international studies, although prevalence varies widely likely due to methodological and geographic differences: 24.8% ESBL-producing *Enterobacterales* in hospital and municipal wastewater in a meta-analysis (Zaatout et al., 2021); 95% in municipal wastewater (Gomez-Sanz et al., 2023) and 36.2% in rivers/lakes in Switzerland (Zurfluh et al., 2013); 98% in polluted rivers in Ghana (Banu et al., 2021). Similarly, carbapenemase-producers have been detected with varied prevalence: carbapenemase-producing *Enterobacterales* were found in all municipal WWTP influent and nearby waters in a U.S. study (Mollenkopf et al., 2025), in 33% hospital wastewater in Iran (Khavandi et al., 2024) and in 68.5% of Nairobi River in Kenya (Too et al., 2024). In addition, both ESBL- and carbapenemase-producers are widespread in China’s Tuojiang and Yangtze rivers (Zhang et al., 2020). Regarding the predominance of *bla_CTX-M_* and the presence of *bla_KPC_* and *bla_NDM_* in our study, it aligns with their known global dissemination (Bevan et al., 2017; Wu et al., 2019; Zou et al., 2019; Bush and Bradford, 2020; Gomi et al., 2022; Teixeira et al., 2022; Wasko et al., 2022; Kotzamanidis et al., 2024; Monge-Olivares et al., 2025).

Although metagenomic approaches provide greater sensitivity (Knight et al., 2024), our culture-based methodology enabled phenotypic characterization, identification of resistance genes and detailed molecular analysis within specific strains. In this regard, ESBL- and carbapenemase-producing strains from wastewater overflows and the Suquía River exhibited multidrug-resistant profiles, frequently including co-resistance to ciprofloxacin and trimethoprim-sulfamethoxazole, resembling clinical isolates. Comparable findings have been reported elsewhere: in Colombia, 63.2% *E. coli* isolates were multidrug-resistant (Aristizabal-Hoyos et al., 2019), while in Ireland, up to 53.98% of fecal coliforms exhibited multidrug-resistance, with ciprofloxacin and trimethoprim-sulfamethoxazole among the most frequent co-resistances (Smyth et al., 2020). The identification of ESBL- and carbapenemase-producing *Aeromonas* species in our study is in agreement with their emerging role as environmental reservoirs of clinically relevant resistance genes (Sekizuka et al., 2019; Drk et al., 2023). Notably, these isolates were detected at river sites with high coliform loads and presence of multidrug-resistant bacteria. While a direct link is uncertain, wastewater-impacted riverine environments could facilitate genetic exchange among bacteria that rarely coexist naturally, including *Aeromonas* species and coliform bacteria.

A remarkable finding of our study is that multidrug-resistant bacteria were detected only in the urban stretch of the Suquía River, underscoring Córdoba’s anthropogenic impact. Antibiotic-resistant species found in the river closely matched those from wastewater overflows, supporting their role as a source. Interestingly, our finding of ciprofloxacin as the most frequent co-resistance in ESBL producers, is consistent with the reported presence of subinhibitory levels of cephalosporins and fluoroquinolones in the Suquía River (Valdes et al., 2021). It has been shown that sustained low levels of antibiotics in the environment can facilitate selection of resistance (Chukwu et al., 2023). In this context, wastewater overflows may act as vectors of coliform bacteria, antibiotic-resistant bacteria of critical importance (ESBL- and carbapenemase-producing *Enterobacterales*), and likely also subinhibitory concentrations of antibiotics into the river via the stormwater system. This aligns with other studies identifying stormwater systems as pathways for anthropogenic contamination of rivers (Eramo et al., 2017; Almakki et al., 2019). The complex mixture transported by these waters likely creates microenvironments that facilitate microbial interactions, gene exchange and selection of antibiotic resistance.

We used WGS to characterize all carbapenemase-producing bacteria from wastewater and the Suquía River, revealing ample resistomes. All KPC-producing strains carried *bla_KPC-2_*, while the NDM-producer carried *bla_NDM-1_*, alleles widespread in clinical strains globally (David et al., 2019; Estabrook et al., 2023; Faccone et al., 2023). Interestingly, the KPC-producing *A. caviae* strain (4.5Aer) from the Suquía River carried only 8 resistance genes and exhibited a novel MLST profile, consistent with an environmental autochthonous origin. Even though in most strains the *bla_KPC-2_* gene was found within the clinically-recognized “Non-Tn4401 variant 1a” transposon (Gomez et al., 2011; Reyes et al., 2020), the unique arrangement found for this gene in *E. kobei* (1.4Eko) from the Suquía River supports the existence of distinct environmental variants. Overall, WGS analysis provides molecular confirmation that carbapenemase-producing bacteria carrying mobile genetic elements identical to those found in clinical strains, are present in wastewater overflows discharged into public areas of Córdoba and in the Suquía River influenced by this discharge.

Regarding the virulome, these isolates carry virulence genes like those in clinical strains (Morgado et al., 2024; Rahmat Ullah et al., 2024), indicating significant virulent potential. Notably, Type III secretion system genes were found in a KPC-producing *C. freundii* wastewater strain (10Cfr) of high-risk clone ST22, a lineage already associated with carbapenemase production, environmental reservoirs, and hospital outbreaks (Jolivet et al., 2021; Riccobono et al., 2023).

Bioinformatic analyses showed all carbapenemase-producing strains carry multiple plasmids, with carbapenemase genes predicted to be plasmid-borne. In two cases, the carbapenemase gene was on the same contig as a plasmid replicon: *bla_KPC-2_* with IncU in *E. kobei* (1.4Eko) from the Suquía River, and *bla_NDM-1_* with IncA/C2 in *E. hormaechei* (34Eho) from wastewater. IncU plasmids, which have broad host range and low copy number and are proposed to form a single incompatibility group with IncP6 plasmids (Haines et al., 2006; Rozwandowicz et al., 2018), are rarely reported in KPC-producing strains but have been found in both clinical and environmental isolates (Rozwandowicz et al., 2018; Wu et al., 2021). IncA/C plasmids, first identified in the 1970s in multidrug-resistant fish pathogens (Aoki et al., 1971), efficiently spread resistance genes among *Enterobacterales* (Fricke et al., 2009; Lindsey et al., 2011). Recently, they have gained prominence as major vectors in the plasmid-mediated dissemination of *bla_NDM-1_* (Kopotsa et al., 2019). Consistently, the *bla_NDM-1_*–carrying *E. hormaechei* (34Eho) transferred the carbapenemase in conjugation assays, and genomic analysis predicts *bla_NDM-1_* on a conjugative IncA/C2 megaplasmid (Martino et al., 2019).

One of the most significant findings of our study is the genetic structure analysis of carbapenemase-containing contigs, particularly in strains from the Suquía River. In both *E. kobei* (1.4Eko) and *A. caviae* (4.5Aer), the *bla_KPC-2_* gene was located on plasmidic contigs combining clinically associated resistance elements with adjacent regions nearly identical to *Aeromonas* plasmids lacking antibiotic resistance genes. This genetic architecture suggests that resistance elements typically associated with clinical settings have become incorporated into environmental plasmids, most likely as a result of gene exchange between antibiotic-resistant coliforms of anthropogenic origin and native river microbiota. Such events may enable the transfer and persistence of clinically significant resistance determinants in species well adapted to free-living survival in riverine habitats.

Among the limitations of our study, the sampling was restricted to various times and locations, which may not fully capture seasonal variations or the complete temporal dynamics of antimicrobial resistance in the studied environments. In addition, the use of culture-based methods likely underestimated the overall diversity of resistance genes compared to metagenomic approaches and excluded non-culturable bacteria. It is also important to note that our evidence for genetic exchange is indirect, inferred from genetic structures in which clinically recognized elements are adjacent to sequences highly similar to *Aeromonas* plasmids. Finally, as risk assessment was beyond the scope of this study, exposure risks for human populations to these environmental sources remain to be determined.

To our knowledge, this study provides the first report of pollution by ESBL- and carbapenemase-producing bacteria in urban wastewater overflows from public streets in a major Argentine city and constitutes one of the few such investigations in Latin America. The data show that sewage overflows in public areas of the city carry coliform bacteria resistant to critically important antibiotics, making them an AMR hotspot and a significant factor in the dissemination of multidrug-resistant bacteria across urban and peri-urban aquatic environments. This scenario is relevant to other cities, particularly in developing countries, where wastewater overflows are common. Thus, expanding research on environmental antimicrobial resistance is essential to inform effective control and prevention strategies. In an interconnected world, coordinated regional actions and a comprehensive, multisectoral One Health approach, along with increased awareness among healthcare professionals and the public, are key to preserving antibiotic efficacy amid the global antimicrobial resistance crisis.

## Data Availability

The datasets presented in this study can be found in online repositories. The repositories can be found at: https://www.ncbi.nlm.nih.gov/ (accession numbers: JBMHEC000000000, JBMHED000000000, JBMHEE000000000, JBMHEF000000000, JBMHEG000000000 and JBMHEH000000000).

## References

[ref1] Almakki A. Jumas-Bilak E. Marchandin H. Licznar-Fajardo P. (2019). Antibiotic resistance in urban runoff. Sci. Total Environ. 667, 64–76. doi: 10.1016/j.scitotenv.2019.02.183, PMID: 30826682

[ref2] Andreopoulos W. B. Geller A. M. Lucke M. Balewski J. Clum A. Ivanova N. N.. (2022). Deeplasmid: deep learning accurately separates plasmids from bacterial chromosomes. Nucleic Acids Res. 50:e17. doi: 10.1093/nar/gkab1115, PMID: 34871418 PMC8860608

[ref3] Antimicrobial Resistance C. (2022). Global burden of bacterial antimicrobial resistance in 2019: a systematic analysis. Lancet 399, 629–655. doi: 10.1016/S0140-6736(21)02724-0, PMID: 35065702 PMC8841637

[ref4] Aoki T. Egusa S. Kimura T. Watanabe T. (1971). Detection of R factors in naturally occurring *Aeromonas salmonicida* strains. Appl. Microbiol. 22, 716–717. doi: 10.1128/am.22.4.716-717.1971, PMID: 4108649 PMC376391

[ref5] Aristizabal-Hoyos A. M. Rodriguez E. A. Arias L. Jimenez J. N. (2019). High clonal diversity of multidrug-resistant and extended spectrum beta-lactamase-producing *Escherichia coli* in a wastewater treatment plant. J. Environ. Manag. 245, 37–47. doi: 10.1016/j.jenvman.2019.05.073, PMID: 31150908

[ref6] Banu R. A. Alvarez J. M. Reid A. J. Enbiale W. Labi A. K. Ansa E. D. O.. (2021). Extended spectrum beta-lactamase *Escherichia coli* in river waters collected from two cities in Ghana, 2018-2020. Trop. Med. Infect. Dis. 6:105. doi: 10.3390/tropicalmed6020105, PMID: 34203078 PMC8293421

[ref7] Bevan E. R. Jones A. M. Hawkey P. M. (2017). Global epidemiology of CTX-M beta-lactamases: temporal and geographical shifts in genotype. J. Antimicrob. Chemother. 72, 2145–2155. doi: 10.1093/jac/dkx146, PMID: 28541467

[ref8] Biez L. Bonnin R. A. Emeraud C. Birer A. Jousset A. B. Naas T.. (2023). Nationwide molecular epidemiology of carbapenemase-producing *Citrobacter* spp. in France in 2019 and 2020. mSphere 8:e0036623. doi: 10.1128/msphere.00366-23, PMID: 37815363 PMC10732076

[ref9] Bolger A. M. Lohse M. Usadel B. (2014). Trimmomatic: a flexible trimmer for Illumina sequence data. Bioinformatics 30, 2114–2120. doi: 10.1093/bioinformatics/btu170, PMID: 24695404 PMC4103590

[ref10] Bush K. (2013). Carbapenemases: partners in crime. J. Glob. Antimicrob. Resist. 1, 7–16. doi: 10.1016/j.jgar.2013.01.005, PMID: 27873609

[ref11] Bush K. (2018). Past and present perspectives on beta-lactamases. Antimicrob. Agents Chemother. 62:e01076-18. doi: 10.1128/AAC.01076-18 30061284 PMC6153792

[ref12] Bush K. Bradford P. A. (2020). Epidemiology of beta-lactamase-producing pathogens. Clin. Microbiol. Rev. 33:e00047-19. doi: 10.1128/CMR.00047-19 32102899 PMC7048014

[ref13] Camacho C. Coulouris G. Avagyan V. Ma N. Papadopoulos J. Bealer K.. (2009). BLAST+: architecture and applications. BMC Bioinformatics 10:421. doi: 10.1186/1471-2105-10-421, PMID: 20003500 PMC2803857

[ref14] Carattoli A. Zankari E. Garcia-Fernandez A. Voldby Larsen M. Lund O. Villa L.. (2014). *In silico* detection and typing of plasmids using PlasmidFinder and plasmid multilocus sequence typing. Antimicrob. Agents Chemother. 58, 3895–3903. doi: 10.1128/AAC.02412-14, PMID: 24777092 PMC4068535

[ref15] Chukwu K. B. Abafe O. A. Amoako D. G. Ismail A. Essack S. Y. Abia A. L. K. (2023). Impact of environmental sub-inhibitory concentrations of antibiotics, heavy metals, and biocides on the emergence of tolerance and effects on the mutant selection window in *E. coli*. Microorganisms 11:2265. doi: 10.3390/microorganisms11092265, PMID: 37764108 PMC10535725

[ref16] CLSI (2016). Methods for Antimicrobial dilution and disk susceptibility testing of infrequently isolated or fastidious Bacteria. 3rd Edn. Wayne, PA: CLSI guideline M45-ED3.

[ref17] CLSI (2024). Clinical and laboratory standards institute (CLSI). Performance standards for Antimicrobial susceptibility testing. 34th Edn. Wayne PA: CLSI supplement M100.

[ref18] Czatzkowska M. Wolak I. Harnisz M. Korzeniewska E. (2022). Impact of anthropogenic activities on the dissemination of ARGs in the environment-A review. Int. J. Environ. Res. Public Health 19:12853. doi: 10.3390/ijerph191912853, PMID: 36232152 PMC9564893

[ref19] David S. Reuter S. Harris S. R. Glasner C. Feltwell T. Argimon S.. (2019). Epidemic of carbapenem-resistant *Klebsiella pneumoniae* in Europe is driven by nosocomial spread. Nat. Microbiol. 4, 1919–1929. doi: 10.1038/s41564-019-0492-8, PMID: 31358985 PMC7244338

[ref20] D'Costa V. M. King C. E. Kalan L. Morar M. Sung W. W. Schwarz C.. (2011). Antibiotic resistance is ancient. Nature 477, 457–461. doi: 10.1038/nature10388, PMID: 21881561

[ref21] Dong W. Fan X. Guo Y. Wang S. Jia S. Lv N.. (2024). An expanded database and analytical toolkit for identifying bacterial virulence factors and their associations with chronic diseases. Nat. Commun. 15:8084. doi: 10.1038/s41467-024-51864-y, PMID: 39278950 PMC11402979

[ref22] Dong D. Mi Z. Li D. Gao M. Jia N. Li M.. (2020). Novel IncR/IncP6 hybrid plasmid pCRE3-KPC recovered from a clinical KPC-2-producing *Citrobacter braakii* isolate. mSphere 5:e00891-19. doi: 10.1128/mSphere.00891-19, PMID: 32213624 PMC7096625

[ref23] Drieux L. Brossier F. Sougakoff W. Jarlier V. (2008). Phenotypic detection of extended-spectrum beta-lactamase production in Enterobacteriaceae: review and bench guide. Clin. Microbiol. Infect. 14, 90–103. doi: 10.1111/j.1469-0691.2007.01846.x 18154532

[ref24] Drk S. Puljko A. Dzelalija M. Udikovic-Kolic N. (2023). Characterization of third generation cephalosporin- and Carbapenem-resistant Aeromonas isolates from municipal and hospital wastewater. Antibiotics (Basel) 12:513. doi: 10.3390/antibiotics12030513, PMID: 36978380 PMC10044312

[ref25] Eramo A. Delos Reyes H. Fahrenfeld N. L. (2017). Partitioning of antibiotic Resistance genes and fecal indicators varies intra and inter-storm during combined sewer overflows. Front. Microbiol. 8:2024. doi: 10.3389/fmicb.2017.02024, PMID: 29104562 PMC5655003

[ref26] Estabrook M. Muyldermans A. Sahm D. Pierard D. Stone G. Utt E. (2023). Epidemiology of Resistance determinants identified in Meropenem-nonsusceptible Enterobacterales collected as part of a global surveillance study, 2018 to 2019. Antimicrob. Agents Chemother. 67:e0140622. doi: 10.1128/aac.01406-22, PMID: 37074173 PMC10190273

[ref27] EUCAST (2024). European committee on Antimicrobial susceptibility testing (EUCAST). Breakpoint tables for interpretation of MICs and zone diameters, version 14.0, 2024.

[ref28] Faccone D. Gomez S. A. de Mendieta J. M. Sanz M. B. Echegorry M. Albornoz E.. (2023). Emergence of hyper-epidemic clones of Enterobacterales clinical isolates co-producing KPC and Metallo-Beta-lactamases during the COVID-19 pandemic. Pathogens 12:479. doi: 10.3390/pathogens12030479, PMID: 36986401 PMC10052147

[ref29] Feldgarden M. Brover V. Gonzalez-Escalona N. Frye J. G. Haendiges J. Haft D. H.. (2021). AMRFinderPlus and the reference gene catalog facilitate examination of the genomic links among antimicrobial resistance, stress response, and virulence. Sci. Rep. 11:12728. doi: 10.1038/s41598-021-91456-0, PMID: 34135355 PMC8208984

[ref30] Fernandez-Bravo A. Figueras M. J. (2020). An update on the genus Aeromonas: taxonomy, epidemiology, and pathogenicity. Microorganisms 8:129. doi: 10.3390/microorganisms8010129, PMID: 31963469 PMC7022790

[ref31] Forsberg K. J. Reyes A. Wang B. Selleck E. M. Sommer M. O. Dantas G. (2012). The shared antibiotic resistome of soil bacteria and human pathogens. Science 337, 1107–1111. doi: 10.1126/science.1220761, PMID: 22936781 PMC4070369

[ref32] Fricke W. F. Welch T. J. McDermott P. F. Mammel M. K. LeClerc J. E. White D. G.. (2009). Comparative genomics of the IncA/C multidrug resistance plasmid family. J. Bacteriol. 191, 4750–4757. doi: 10.1128/JB.00189-09, PMID: 19482926 PMC2715731

[ref33] Ghiglione B. Haim M. S. Penzotti P. Brunetti F. D A G G. Di Conza J.. (2021). Characterization of emerging pathogens carrying Bla(KPC-2) gene in IncP-6 plasmids isolated from urban sewage in Argentina. Front. Cell. Infect. Microbiol. 11:722536. doi: 10.3389/fcimb.2021.722536, PMID: 34504809 PMC8421773

[ref34] Gomez S. A. Pasteran F. G. Faccone D. Tijet N. Rapoport M. Lucero C.. (2011). Clonal dissemination of *Klebsiella pneumoniae* ST258 harbouring KPC-2 in Argentina. Clin. Microbiol. Infect. 17, 1520–1524. doi: 10.1111/j.1469-0691.2011.03600.x, PMID: 21851480

[ref35] Gomez-Sanz E. Bagutti C. Roth J. A. Alt Hug M. Garcia-Martin A. B. Maurer Pekerman L.. (2023). Spatiotemporal dissemination of ESBL-producing Enterobacterales in municipal sewer systems: a prospective, longitudinal study in the city of Basel, Switzerland. Front. Microbiol. 14:1174336. doi: 10.3389/fmicb.2023.1174336, PMID: 37250050 PMC10213686

[ref36] Gomi R. Yamamoto M. Tanaka M. Matsumura Y. (2022). Chromosomal integration of Bla (CTX-M) genes in diverse *Escherichia coli* isolates recovered from river water in Japan. Curr. Res. Microb. Sci. 3:100144. doi: 10.1016/j.crmicr.2022.100144, PMID: 35909619 PMC9325909

[ref37] Haines A. S. Cheung M. Thomas C. M. (2006). Evidence that IncG (IncP-6) and IncU plasmids form a single incompatibility group. Plasmid 55, 210–215. doi: 10.1016/j.plasmid.2005.11.003, PMID: 16439021

[ref38] Hammoudi Halat D. Ayoub Moubareck C. (2020). The current burden of Carbapenemases: review of significant properties and dissemination among gram-negative Bacteria. Antibiotics (Basel) 9:186. doi: 10.3390/antibiotics9040186, PMID: 32316342 PMC7235769

[ref39] Jolivet S. Couturier J. Vuillemin X. Gouot C. Nesa D. Adam M.. (2021). Outbreak of OXA-48-producing Enterobacterales in a haematological ward associated with an uncommon environmental reservoir, France, 2016 to 2019. Euro Surveill. 26:2000118. doi: 10.2807/1560-7917.ES.2021.26.21.2000118, PMID: 34047273 PMC8161731

[ref40] Jolley K. A. Bliss C. M. Bennett J. S. Bratcher H. B. Brehony C. Colles F. M.. (2012). Ribosomal multilocus sequence typing: universal characterization of bacteria from domain to strain. Microbiology (Reading) 158, 1005–1015. doi: 10.1099/mic.0.055459-0, PMID: 22282518 PMC3492749

[ref41] Jolley K. A. Bray J. E. Maiden M. C. J. (2018). Open-access bacterial population genomics: BIGSdb software, the PubMLST.org website and their applications. Wellcome Open Res. 3:124. doi: 10.12688/wellcomeopenres.14826.1, PMID: 30345391 PMC6192448

[ref42] Jones D. C. LaMartina E. L. Lewis J. R. Dahl A. J. Nadig N. Szabo A.. (2023). One health and Global Health view of Antimicrobial susceptibility through the "eye" of Aeromonas: systematic review and Meta-analysis. Int. J. Antimicrob. Agents 62:106848. doi: 10.1016/j.ijantimicag.2023.106848, PMID: 37201798 PMC10524465

[ref43] Khavandi S. Habibzadeh N. Hasani K. Sardari M. Arzanlou M. (2024). Carbapenem-resistant Enterobacterales in wastewater resources and healthy carriers: A survey in Iran. J. Water Health 22, 1053–1063. doi: 10.2166/wh.2024.041, PMID: 38935456

[ref44] Knight M. E. Webster G. Perry W. B. Baldwin A. Rushton L. Pass D. A.. (2024). National-scale antimicrobial resistance surveillance in wastewater: A comparative analysis of HT qPCR and metagenomic approaches. Water Res. 262:121989. doi: 10.1016/j.watres.2024.121989, PMID: 39018584

[ref45] Kopotsa K. Osei Sekyere J. Mbelle N. M. (2019). Plasmid evolution in carbapenemase-producing Enterobacteriaceae: a review. Ann. N. Y. Acad. Sci. 1457, 61–91. doi: 10.1111/nyas.14223, PMID: 31469443

[ref46] Kotzamanidis C. Malousi A. Paraskeva A. Vafeas G. Giantzi V. Hatzigiannakis E.. (2024). River waters in Greece: A reservoir for clinically relevant extended-spectrum-beta-lactamases-producing *Escherichia coli*. Sci. Total Environ. 941:173554. doi: 10.1016/j.scitotenv.2024.173554, PMID: 38823724

[ref47] Larsson D. G. J. Andremont A. Bengtsson-Palme J. Brandt K. K. de Roda Husman A. M. Fagerstedt P.. (2018). Critical knowledge gaps and research needs related to the environmental dimensions of antibiotic resistance. Environ. Int. 117, 132–138. doi: 10.1016/j.envint.2018.04.041, PMID: 29747082

[ref48] Larsson D. G. J. Flach C. F. (2022). Antibiotic resistance in the environment. Nat. Rev. Microbiol. 20, 257–269. doi: 10.1038/s41579-021-00649-x, PMID: 34737424 PMC8567979

[ref49] LaVoz (2017). El municipio admite 30 desbordes cloacales promedio cada día. [Online]. La Voz del Interior. Available online at: https://www.lavoz.com.ar/ciudadanos/el-municipio-admite-30-desbordes-cloacales-promedio-cada-dia/

[ref50] LaVoz (2021). Desborde cloacal en barrio Alberdi: se agrava la crisis ambiental. [Online]. Available online at: https://www.lavoz.com.ar/ciudadanos/desborde-cloacal-en-barrio-alberdi-se-agrava-la-crisis-ambiental/ (Accessed July 7, 2025).

[ref51] Laxminarayan R. Duse A. Wattal C. Zaidi A. K. Wertheim H. F. Sumpradit N.. (2013). Antibiotic resistance-the need for global solutions. Lancet Infect. Dis. 13, 1057–1098. doi: 10.1016/S1473-3099(13)70318-9, PMID: 24252483

[ref52] Lee J. Ju F. Beck K. Burgmann H. (2023). Differential effects of wastewater treatment plant effluents on the antibiotic resistomes of diverse river habitats. ISME J. 17, 1993–2002. doi: 10.1038/s41396-023-01506-w, PMID: 37684524 PMC10579368

[ref53] Lindsey R. L. Frye J. G. Fedorka-Cray P. J. Meinersmann R. J. (2011). Microarray-based analysis of IncA/C plasmid-associated genes from multidrug-resistant *Salmonella enterica*. Appl. Environ. Microbiol. 77, 6991–6999. doi: 10.1128/AEM.00567-11, PMID: 21841024 PMC3187115

[ref54] Lipps W. C. Baxter T. E. (2023). Standard methods for the examination of water and wastewater. 24th Edn. Washington, D.C., USA: American Public Health Association, American Water Works Association and Water Environment Federation.

[ref55] Liu B. Zheng D. Zhou S. Chen L. Yang J. (2022). VFDB 2022: a general classification scheme for bacterial virulence factors. Nucleic Acids Res. 50, D912–D917. doi: 10.1093/nar/gkab1107, PMID: 34850947 PMC8728188

[ref56] Manaia C. M. Macedo G. Fatta-Kassinos D. Nunes O. C. (2016). Antibiotic resistance in urban aquatic environments: can it be controlled? Appl. Microbiol. Biotechnol. 100, 1543–1557. doi: 10.1007/s00253-015-7202-0, PMID: 26649735

[ref57] Martino F. Tijet N. Melano R. Petroni A. Heinz E. De Belder D.. (2019). Isolation of five Enterobacteriaceae species harbouring blaNDM-1 and mcr-1 plasmids from a single paediatric patient. PLoS One 14:e0221960. doi: 10.1371/journal.pone.0221960, PMID: 31498841 PMC6733481

[ref58] Mikheenko A. Prjibelski A. Saveliev V. Antipov D. Gurevich A. (2018). Versatile genome assembly evaluation with QUAST-LG. Bioinformatics 34, i142–i150. doi: 10.1093/bioinformatics/bty266, PMID: 29949969 PMC6022658

[ref59] Mollenkopf D. F. Lee S. Ballash G. A. Sullivan S. M. P. Lee J. Wittum T. E. (2025). Carbapenemase-producing Enterobacterales and their carbapenemase genes are stably recovered across the wastewater-watershed ecosystem nexus. Sci. Total Environ. 975:179241. doi: 10.1016/j.scitotenv.2025.179241, PMID: 40174253

[ref60] Monge-Olivares L. Penalva G. Pulido M. R. Garrudo L. Angel Doval M. Ballesta S.. (2025). Quantitative study of ESBL and carbapenemase producers in wastewater treatment plants in Seville, Spain: a culture-based detection analysis of raw and treated water. Water Res. 281:123706. doi: 10.1016/j.watres.2025.123706 40311350

[ref61] Morgado S. Fonseca E. Freitas F. Caldart R. Vicente A. C. (2024). In-depth analysis of *Klebsiella aerogenes* resistome, virulome and plasmidome worldwide. Sci. Rep. 14:6538. doi: 10.1038/s41598-024-57245-1, PMID: 38503805 PMC10951357

[ref62] Morosini M. I. (2017). The endless increase of antibiotic resistance in Enterobacteriaceae and the activity of new compounds to face the challenge. Enferm. Infecc. Microbiol. Clin. 35, 477–479. doi: 10.1016/j.eimc.2017.08.007, PMID: 28964321

[ref63] Mutuku C. Gazdag Z. Melegh S. (2022). Occurrence of antibiotics and bacterial resistance genes in wastewater: resistance mechanisms and antimicrobial resistance control approaches. World J. Microbiol. Biotechnol. 38:152. doi: 10.1007/s11274-022-03334-0, PMID: 35781751 PMC9250919

[ref64] O’Neill J. (2016). Tackling drug-resistant infections globally: Final report and recommendations. Review on Antimicrobial Resistance. London, UK: Wellcome Trust and HM Government.

[ref65] Parks D. H. Imelfort M. Skennerton C. T. Hugenholtz P. Tyson G. W. (2015). CheckM: assessing the quality of microbial genomes recovered from isolates, single cells, and metagenomes. Genome Res. 25, 1043–1055. doi: 10.1101/gr.186072.114, PMID: 25977477 PMC4484387

[ref66] Perez F. Bonomo R. A. (2019). Carbapenem-resistant Enterobacteriaceae: global action required. Lancet Infect. Dis. 19, 561–562. doi: 10.1016/S1473-3099(19)30210-5, PMID: 31047851

[ref67] Prjibelski A. Antipov D. Meleshko D. Lapidus A. Korobeynikov A. (2020). Using SPAdes de novo assembler. Curr. Protoc. Bioinform. 70:e102. doi: 10.1002/cpbi.102, PMID: 32559359

[ref68] Queipo-Ortuno M. I. De Dios Colmenero J. Macias M. Bravo M. J. Morata P. (2008). Preparation of bacterial DNA template by boiling and effect of immunoglobulin G as an inhibitor in real-time PCR for serum samples from patients with brucellosis. Clin. Vaccine Immunol. 15, 293–296. doi: 10.1128/CVI.00270-07, PMID: 18077622 PMC2238042

[ref69] Rahmat Ullah S. Irum S. Mahnoor I. Ismatullah H. Mumtaz M. Andleeb S.. (2024). Exploring the resistome, virulome, and mobilome of multidrug-resistant *Klebsiella pneumoniae* isolates: deciphering the molecular basis of carbapenem resistance. BMC Genomics 25:408. doi: 10.1186/s12864-024-10139-y, PMID: 38664636 PMC11044325

[ref70] Reyes J. A. Melano R. Cardenas P. A. Trueba G. (2020). Mobile genetic elements associated with carbapenemase genes in south American Enterobacterales. Braz. J. Infect. Dis. 24, 231–238. doi: 10.1016/j.bjid.2020.03.002, PMID: 32325019 PMC9392046

[ref71] Riccobono E. Salvetti S. Coppi M. Montenora I. Di Pilato V. Rossolini G. M. (2023). *Citrobacter freundii* resistant to novel beta-lactamase inhibitor combinations and cefiderocol, co-producing class A, B and D carbapenemases encoded by transferable plasmids. J. Antimicrob. Chemother. 78, 1677–1682. doi: 10.1093/jac/dkad150, PMID: 37207353

[ref72] Robertson J. Nash J. H. E. (2018). MOB-suite: software tools for clustering, reconstruction and typing of plasmids from draft assemblies. Microb. Genom. 4:e000206. doi: 10.1099/mgen.0.000206, PMID: 30052170 PMC6159552

[ref73] Roca I. Akova M. Baquero F. Carlet J. Cavaleri M. Coenen S.. (2015). The global threat of antimicrobial resistance: science for intervention. New Microbes New Infect. 6, 22–29. doi: 10.1016/j.nmni.2015.02.007, PMID: 26029375 PMC4446399

[ref74] Rozwandowicz M. Brouwer M. S. M. Fischer J. Wagenaar J. A. Gonzalez-Zorn B. Guerra B.. (2018). Plasmids carrying antimicrobial resistance genes in Enterobacteriaceae. J. Antimicrob. Chemother. 73, 1121–1137. doi: 10.1093/jac/dkx488, PMID: 29370371

[ref75] Sayers E. W. Beck J. Bolton E. E. Brister J. R. Chan J. Connor R.. (2025). Database resources of the National Center for biotechnology information in 2025. Nucleic Acids Res. 53, D20–D29. doi: 10.1093/nar/gkae979, PMID: 39526373 PMC11701734

[ref76] Schwengers O. Jelonek L. Dieckmann M. A. Beyvers S. Blom J. Goesmann A. (2021). Bakta: rapid and standardized annotation of bacterial genomes via alignment-free sequence identification. Microb. Genom. 7:000685. doi: 10.1099/mgen.0.000685 34739369 PMC8743544

[ref77] Sekizuka T. Inamine Y. Segawa T. Hashino M. Yatsu K. Kuroda M. (2019). Potential KPC-2 carbapenemase reservoir of environmental Aeromonas hydrophila and *Aeromonas caviae* isolates from the effluent of an urban wastewater treatment plant in Japan. Environ. Microbiol. Rep. 11, 589–597. doi: 10.1111/1758-2229.12772, PMID: 31106978 PMC6851574

[ref78] Shimoyama Y. (2024). pyGenomeViz: A genome visualization python package for comparative genomics. Available online at: https://github.com/moshi4/pyGenomeViz (Accessed May 25, 2025).

[ref79] Siguier P. Perochon J. Lestrade L. Mahillon J. Chandler M. (2006). ISfinder: the reference Centre for bacterial insertion sequences. Nucleic Acids Res. 34, D32–D36. doi: 10.1093/nar/gkj014, PMID: 16381877 PMC1347377

[ref80] Silver L. L. (2011). Challenges of antibacterial discovery. Clin. Microbiol. Rev. 24, 71–109. doi: 10.1128/CMR.00030-10, PMID: 21233508 PMC3021209

[ref81] Singer A. C. Shaw H. Rhodes V. Hart A. (2016). Review of Antimicrobial Resistance in the environment and its relevance to environmental regulators. Front. Microbiol. 7:1728. doi: 10.3389/fmicb.2016.01728, PMID: 27847505 PMC5088501

[ref82] Smyth C. O'Flaherty A. Walsh F. Do T. T. (2020). Antibiotic resistant and extended-spectrum beta-lactamase producing faecal coliforms in wastewater treatment plant effluent. Environ. Pollut. 262:114244. doi: 10.1016/j.envpol.2020.114244 32146363

[ref83] Teixeira P. Tacao M. Henriques I. (2022). Occurrence and distribution of Carbapenem-resistant Enterobacterales and carbapenemase genes along a highly polluted hydrographic basin. Environ. Pollut. 300:118958. doi: 10.1016/j.envpol.2022.118958, PMID: 35131334

[ref84] Too R. J. Kariuki S. M. Gitao G. C. Bebora L. C. Mollenkopf D. F. Wittum T. E. (2024). Carbapenemase-producing bacteria recovered from Nairobi River, Kenya surface water and from nearby anthropogenic and zoonotic sources. PLoS One 19:e0310026. doi: 10.1371/journal.pone.0310026, PMID: 39541397 PMC11563437

[ref85] Valdes M. E. Santos L. Rodriguez Castro M. C. Giorgi A. Barcelo D. Rodriguez-Mozaz S.. (2021). Distribution of antibiotics in water, sediments and biofilm in an urban river (Cordoba, Argentina, LA). Environ. Pollut. 269:116133. doi: 10.1016/j.envpol.2020.116133, PMID: 33316497

[ref86] Wang J. Chen Y. Cai P. Gao Q. Zhong H. Sun W.. (2022). Impacts of municipal wastewater treatment plant discharge on microbial community structure and function of the receiving river in northwest Tibetan plateau. J. Hazard. Mater. 423:127170. doi: 10.1016/j.jhazmat.2021.127170, PMID: 34537645

[ref87] Wasko I. Kozinska A. Kotlarska E. Baraniak A. (2022). Clinically relevant beta-lactam resistance genes in wastewater treatment plants. Int. J. Environ. Res. Public Health 19:13829. doi: 10.3390/ijerph192113829 36360709 PMC9657204

[ref88] Weber R. E. Pietsch M. Fruhauf A. Pfeifer Y. Martin M. Luft D.. (2019). IS26-mediated transfer of Bla (NDM-1) as the Main route of Resistance transmission during a polyclonal, multispecies outbreak in a German hospital. Front. Microbiol. 10:2817. doi: 10.3389/fmicb.2019.02817, PMID: 31921015 PMC6929489

[ref89] White A. Hughes J. M. (2019). Critical importance of a one health approach to antimicrobial resistance. EcoHealth 16, 404–409. doi: 10.1007/s10393-019-01415-5, PMID: 31250160

[ref90] WHO (2024). WHO bacterial priority pathogens list, 2024: Bacterial pathogens of public health importance to guide research, development and strategies to prevent and control antimicrobial resistance. Licence: CC BY-NC-SA 3.0 IGO. [Online]. Available online at: https://www.who.int/publications/i/item/9789240093461 (Accessed April 23, 2025).

[ref91] Wu W. Feng Y. Tang G. Qiao F. McNally A. Zong Z. (2019). NDM Metallo-beta-lactamases and their bacterial producers in health care settings. Clin. Microbiol. Rev. 32:e00115-18. doi: 10.1128/CMR.00115-18, PMID: 30700432 PMC6431124

[ref92] Wu W. Lu L. Fan W. Chen C. Jin D. Pan H.. (2021). Complete genome sequences of two novel KPC-2-producing IncU multidrug-resistant plasmids from international high-risk clones of *Escherichia coli* in China. Front. Microbiol. 12:698478. doi: 10.3389/fmicb.2021.698478, PMID: 34367098 PMC8335537

[ref93] Zaatout N. Bouras S. Slimani N. (2021). Prevalence of extended-spectrum beta-lactamase (ESBL)-producing Enterobacteriaceae in wastewater: a systematic review and meta-analysis. J. Water Health 19, 705–723. doi: 10.2166/wh.2021.112, PMID: 34665765

[ref94] Zhang R. Gu J. Wang X. Li Y. Zhang K. Yin Y.. (2018). Contributions of the microbial community and environmental variables to antibiotic resistance genes during co-composting with swine manure and cotton stalks. J. Hazard. Mater. 358, 82–91. doi: 10.1016/j.jhazmat.2018.06.052, PMID: 29990821

[ref95] Zhang L. Ma X. Luo L. Hu N. Duan J. Tang Z.. (2020). The prevalence and characterization of extended-Spectrum beta-lactamase- and Carbapenemase-producing Bacteria from hospital sewage, treated effluents and receiving Rivers. Int. J. Environ. Res. Public Health 17:1183. doi: 10.3390/ijerph17041183, PMID: 32069792 PMC7068339

[ref96] Zou H. Zheng B. Sun M. Ottoson J. Li Y. Berglund B.. (2019). Evaluating dissemination mechanisms of antibiotic-resistant Bacteria in rural environments in China by using CTX-M-producing *Escherichia coli* as an Indicator. Microb. Drug Resist. 25, 975–984. doi: 10.1089/mdr.2018.0431, PMID: 30942653

[ref97] Zurfluh K. Hachler H. Nuesch-Inderbinen M. Stephan R. (2013). Characteristics of extended-spectrum beta-lactamase- and carbapenemase-producing Enterobacteriaceae isolates from rivers and lakes in Switzerland. Appl. Environ. Microbiol. 79, 3021–3026. doi: 10.1128/AEM.00054-13, PMID: 23455339 PMC3623138

