# Supplementary figures and images for: Urban wastewater overflows as hotspots for dissemination of bacteria producing extended-spectrum β-lactamases and carbapenemases in the Suquía River, Argentina
Source: Front Microbiol. 2025 Sep 24;16:1669531. doi: 10.3389/fmicb.2025.1669531 (PMC12504239; doi:10.3389/fmicb.2025.1669531)

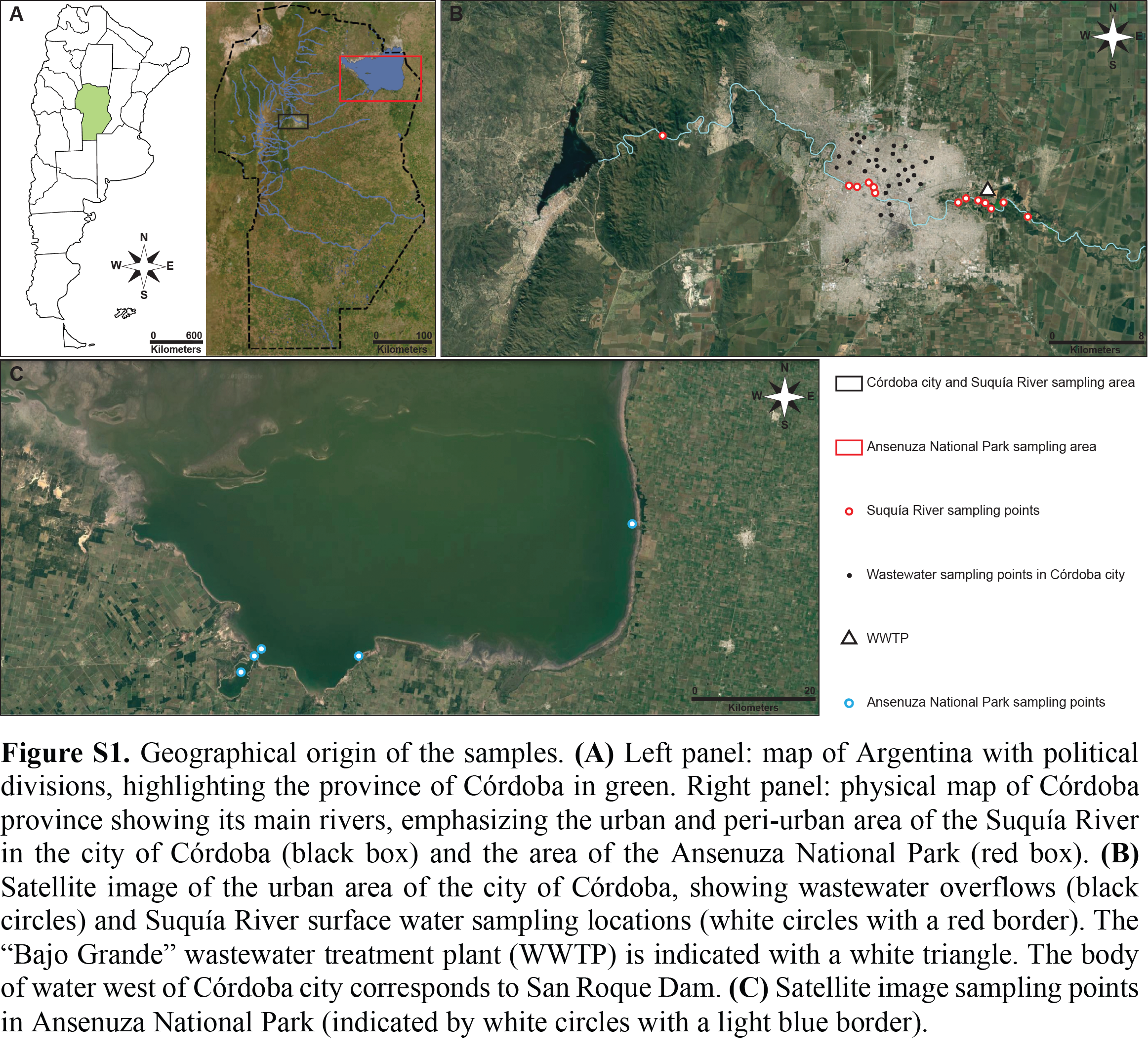

Supplement: Supplementary file 1 [file Image_1.tif]
