# Supplementary material for: Urban wastewater overflows as hotspots for dissemination of bacteria producing extended-spectrum β-lactamases and carbapenemases in the Suquía River, Argentina
Source: Front Microbiol. 2025 Sep 24;16:1669531. doi: 10.3389/fmicb.2025.1669531 (PMC12504239; doi:10.3389/fmicb.2025.1669531)
Supplement: Supplementary file 3 [file Table_2.docx]

| **Sample ID** | **Sample type, origin** | **Strain ID** | **Species**  **ID** | **Resistance phenotype**  **(genetic determinant)** |
| --- | --- | --- | --- | --- |
| AR1 | Wastewater, Córdoba city | 1Eco | *Escherichia coli* | ESBL (other*) |
|  |  | 1Eclo | *Enterobacter cloacae complex* | ESBL (other*) |
| AR2 | Wastewater, Córdoba city | 2Eco | *Escherichia coli* | ESBL (*bla_CTX-M_*) |
| AR5 | Wastewater, Córdoba city | 5Eco1 | *Escherichia coli* | ESBL (*bla_CTX-M_*) |
|  |  | 5Eco2 | *Escherichia coli* | ESBL (other*) |
|  |  | 5Cit | *Citrobacter freundii* | ESBL (other*) |
| AR6 | Wastewater, Córdoba city | 6Eco | *Escherichia coli* | ESBL (*bla_CTX-M_*) |
| AR7 | Wastewater, Córdoba city | 7Eco1 | *Escherichia coli* | ESBL (other*) |
|  |  | 7Eco2 | *Escherichia coli* | ESBL (other*) |
| AR8 | Wastewater, Córdoba city | 8Eco | *Escherichia coli* | ESBL (*bla_CTX-M_*) |
| AR10 | Wastewater, Córdoba city | 10Cfr | *Citrobacter freundii* | Carbapenemase (*bla_KPC_*) |
|  |  | 10Kmi | *Klebsiella michiganensis* | Carbapenemase (*bla_KPC_*) |
| AR11 | Wastewater, Córdoba city | 11Eco | *Escherichia coli* | ESBL (*bla_CTX-M_*) |
| AR14 | Wastewater, Córdoba city | 14Eco | *Escherichia coli* | ESBL (*bla_CTX-M_*) |
| AR16 | Wastewater, Córdoba city | 16Eco | *Escherichia coli* | ESBL (*bla_CTX-M_*) |
|  |  | 16Aer | *Aeromonas hidrophila* | ESBL (other*) |
| AR17 | Wastewater, Córdoba city | 17Eco | *Escherichia coli* | ESBL (*bla_CTX-M_*) |
| AR18 | Wastewater, Córdoba city | 18Kpn | *Klebsiella pneumoniae* | ESBL (*bla_CTX-M_*) |
| AR21 | Wastewater, Córdoba city | 21Eco | *Escherichia coli* | ESBL (*bla_SHV_*) |
| AR24 | Wastewater, Córdoba city | 24Eco1 | *Escherichia coli* | ESBL (*bla_CTX-M_*) |
|  |  | 24Eco2 | *Escherichia coli* | ESBL (*bla_CTX-M_*) |
| AR25 | Wastewater, Córdoba city | 25Eco | *Escherichia coli* | ESBL (other*) |
|  |  | 25Aer | *Aeromonas caviae* | ESBL (*blaVEB*) |
| AR26 | Wastewater, Córdoba city | 26Eco | *Escherichia coli* | ESBL (*bla_CTX-M_*) |
| AR27 | Wastewater, Córdoba city | 27Eclo | *Enterobacter cloacae complex* | ESBL (other*) |
|  |  | 27Eco | *Escherichia coli* | ESBL (*bla_CTX-M_*) |
| AR28 | Wastewater, Córdoba city | 28Eco | *Escherichia coli* | ESBL (*bla_CTX-M_*) |
| AR29 | Wastewater, Córdoba city | 29Eco | *Escherichia coli* | ESBL (other*) |
| AR30 | Wastewater, Córdoba city | 30Eco | *Escherichia coli* | ESBL (*bla_CTX-M_*) |
| AR31 | Wastewater, Córdoba city | 31Kpn | *Klebsiella pneumoniae* | ESBL (*bla_SHV_*) |
|  |  | 31Ero | *Enterobacter roggenkampii* | Carbapenemase, ESBL (*bla_KPC_, bla_GES_*) |
|  |  | 31Eas | *Enterobacter asburiae* | ESBL (*bla_PER_*) |
|  |  | 31Eco | *Escherichia coli* | ESBL (*bla_CTX-M_*) |
| AR32 | Wastewater, Córdoba city | 32Eco | *Escherichia coli* | ESBL (*bla_CTX-M_*) |
| AR33 | Wastewater, Córdoba city | 33Eco | *Escherichia coli* | ESBL (*bla_CTX-M_*) |
| AR34 | Wastewater, Córdoba city | 34Eho | *Enterobacter hormaechei* | Carbapenemase, ESBL (*bla_NDM_, bla_CTX-M_*) |
|  |  | 34Eco | *Escherichia coli* | ESBL (*bla_CTX-M_*) |
|  |  | 34Kpn | *Klebsiella pneumoniae* | ESBL (*bla_SHV_*) |
| RS1.4 | Suquía River, 6 km downstream of WWTP | 1.4Eko | *Enterobacter kobei* | Carbapenemase, ESBL (*bla_KPC_, bla_VEB_*) |
| RS1.5 | Suquía River, 10 km downstream of WWTP | 1.5Cfr | *Citrobacter freundii* | ESBL (other*) |
| RS2.2 | Suquía River, Campo de La Ribera (Córdoba city) | 2.2Kpn | *Klebsiella pneumoniae* | ESBL *(bla_CTX-M_)* |
| RS2.3 | Suquía River, ring road (Córdoba city) | 2.3Eco | *Escherichia coli* | ESBL *(bla_CTX-M_)* |
| RS2.4 | Suquía River, 6 km downstream of WWTP | 2.4Eco | *Escherichia coli* | ESBL *(bla_CTX-M_)* |
| RS2.5 | Suquía River, 10 km downstream of WWTP | 2.5Eco | *Escherichia coli* | ESBL *(bla_CTX-M_)* |
| RS3.1 | Suquía River at WWTP | 3.1Eco | *Escherichia coli* | ESBL *(bla_CTX-M_)* |
|  |  | 3.1Cfr1 | *Citrobacter freundii* | ESBL *(bla_PER_)* |
|  |  | 3.1Eclo2 | *Enterobacter cloacae complex* | ESBL (other*) |
| RS3.2 | Suquía River, 300 m upstream of WWTP | 3.2Eco | *Escherichia coli* | ESBL *(bla_CTX-M_)* |
| RS3.3 | Suquía River, 600 m downstream of WWTP | 3.3Eco | *Escherichia coli* | ESBL *(bla_CTX-M_)* |
|  |  | 3.3Aer | *Aeromonas caviae* | ESBL *(bla_VEB_)* |
| RS4.5 | Suquía River, 10 km downstream of WWTP | 4.5Eco1 | *Escherichia coli* | ESBL *(bla_CTX-M_)* |
|  |  | 4.5Eco1S | *Escherichia coli* | ESBL *(bla_CTX-M_)* |
|  |  | 4.5Ser | *Serratia marcescens* | ESBL *(bla_SHV_)* |
|  |  | 4.5Kpn | *Klebsiella pneumoniae* | ESBL *(bla_SHV_)* |
|  |  | 4.5Aer | *Aeromonas caviae* | Carbapenemase (*bla_KPC_*) |
| RS6P3 | Suquía River, Zippoli bridge (Córdoba city) | 6P3Eco | *Escherichia coli* | ESBL *(bla_CTX-M_)* |
|  |  | 6P3Aer | *Aeromonas caviae* | ESBL *(bla_PER_)* |
| RS7.P4 | Suquía River, Páez Molina Street (Córdoba city) | 7P4Eco | *Escherichia coli* | ESBL *(bla_CTX-M_)* |
| RS6.P7 | Suquía River, 12 de Octubre Street (Córdoba city) | 6P7Eco | *Escherichia coli* | ESBL *(bla_CTX-M_)* |
| RS5 | Suquía River, Isla de los Patos (Córdoba city) | 5Eco | *Escherichia coli* | ESBL *(bla_CTX-M_)* |
|  |  | 5Kpn | *Klebsiella pneumoniae* | ESBL *(bla_CTX-M_)* |
| RS8.5 | Suquía River, 10 km downstream of WWTP | 8.5Eco1 | *Escherichia coli* | ESBL *(bla_CTX-M_)* |
|  |  | 8.5Eco2 | *Escherichia coli* | ESBL *(bla_CTX-M_)* |
|  |  | 8.5Eco3 | *Escherichia coli* | ESBL *(bla_CTX-M_)* |
|  |  | 8.5Eco4 | *Escherichia coli* | ESBL *(bla_CTX-M_)* |

**Table S2.** **Genetic determinants of ESBL and carbapenemases in strains isolated from wastewater and Suquía River.** A variety of carbapenemases (*bla_KPC_, bla_NDM_, bla_VIM_, bla_IMP_, bla_OXA_* ) and ESBLs (*bla_CTX-M_, bla_PER_, bla_SHV_, bla_GES_, bla_VEB_*) were investigated by PCR. *Other: strains with a confirmed ESBL phenotype and negative PCR results for all investigated ESBL determinants.
