# Supplementary material for: Urban wastewater overflows as hotspots for dissemination of bacteria producing extended-spectrum β-lactamases and carbapenemases in the Suquía River, Argentina
Source: Front Microbiol. 2025 Sep 24;16:1669531. doi: 10.3389/fmicb.2025.1669531 (PMC12504239; doi:10.3389/fmicb.2025.1669531)
Supplement: Supplementary file 4 [file Table_3.docx]

**Table S3**.  **Primers used for detection of β-lactamases and 16S RNA genes.**

| **Gene** | **Primer** | **Sequence** | **Amplicon**  (T° annealing) | **Reference** |
| --- | --- | --- | --- | --- |
| *bla_KPC_* | F-kpcHAS | 5´-CAAGGAATATCGTTGATGTCACTG | 932 bp  (55°C) | This work |
|  | R-kpcHAS | 5´-GCCAATAGATGATTTTCAGAGCC |  |  |
| *bla_NDM_* | F-NDM | 5’-AGCACACTTCCTATCTCGAC | 512 bp  (50°C) | (Faccone et al., 2023) |
|  | R-NDM | 5’-GGCGTAGTGCTCAGTGTC |  |  |
| *bla_VIM_* | F-VIM | 5’-AGTGGTGAGTATCCGACAG | 261 bp  (50°C) | (Faccone et al., 2023) |
|  | R-VIM | 5’-ATGAAAGTGCGTGGAGAC |  |  |
| *bla_IMP_* | IMP-UF1 | 5’-GGYGTTTWTGTTCATACWTCKTTYGA | 404 bp  (50°C) | (Faccone et al., 2023) |
|  | IMP-UR1 | 5’-GGYARCCAAACCACTASGTTATCT |  |  |
| *bla_OXA-48/-163_* | OXA48-F | 5’-ATGCGTGTATTAGCCTTATCGG | 763 bp (*bla_OXA-48_*)  775 bp (*bla_OXA-163_*)  (50°C) | (Faccone et al., 2023) |
|  | OXA48-R2 | 5’-TGAGCACTTCTTTTGTGATG |  |  |
| *bla_CTX-M_* | F-CTXM-Fam | 5’-ATGTGCAGYACCAGTAARGTKATGGC | 593 bp  (51°C) | This work |
|  | R-CTXM-Fam | 5’-TGGGTRAARTARGTSACCAGAAYCAGC |  |  |
| *bla_PER_* | F-PER-Fam | 5’-CAATCCCCDYTGTTAAAAGRGCA | 715 bp  (51°C) | This work |
|  | R-PER-Fam | 5’-CGYCCATCAGGCAACAKAATGA |  |  |
| *bla_shv_* | Fshv-OS1 | 5’-TCGGGCCGCGTAGGCATGAT | 626 bp  (65°C) | (Melano et al., 2003) |
|  | Rshv-OS2 | 5’-AGCAGGGCGACAATCCCGCG |  |  |
| *bla_GES_* | GES-FV1 | 5’-TCCCCAAGGAGAGATCGTCG | 478 bp  (52°C) | (Urase et al., 2022) |
|  | GES-R | 5’-CCTCTCAATGGTGTGGGT |  |  |
| *bla_VEB_* | VEB1-F | 5’-CGACTTCCATTTCCCGATGC | 643 bp  (60°C) | (Zafer et al., 2014) |
|  | VEB1-R | 5’-GGACTCTGCAACAAATACGC |  |  |
| 16S rRNA | 16S-F | 5’-GTGCAATATTCCCCACTGCT | 93 bp  (55°C) | (Kieffer et al., 2019) |
|  | 16S-R | 5’-CGATCCCTAGCTGGTCTGAG |  |  |

**References**

Faccone, D., Gomez, S.A., de Mendieta, J.M., Sanz, M.B., Echegorry, M., Albornoz, E., et al. (2023). Emergence of Hyper-Epidemic Clones of Enterobacterales Clinical Isolates Co-Producing KPC and Metallo-Beta-Lactamases during the COVID-19 Pandemic. *Pathogens* 12(3). doi: 10.3390/pathogens12030479.

Kieffer, N., Royer, G., Decousser, J.W., Bourrel, A.S., Palmieri, M., Ortiz De La Rosa, J.M., et al. (2019). mcr-9, an Inducible Gene Encoding an Acquired Phosphoethanolamine Transferase in Escherichia coli, and Its Origin. *Antimicrob Agents Chemother* 63(9). doi: 10.1128/AAC.00965-19.

Melano, R., Corso, A., Petroni, A., Centron, D., Orman, B., Pereyra, A., et al. (2003). Multiple antibiotic-resistance mechanisms including a novel combination of extended-spectrum beta-lactamases in a Klebsiella pneumoniae clinical strain isolated in Argentina. *J Antimicrob Chemother* 52(1)**,** 36-42. doi: 10.1093/jac/dkg281.

Urase, T., Goto, S., and Sato, M. (2022). Monitoring Carbapenem-Resistant Enterobacterales in the Environment to Assess the Spread in the Community. *Antibiotics (Basel)* 11(7). doi: 10.3390/antibiotics11070917.

Zafer, M.M., Al-Agamy, M.H., El-Mahallawy, H.A., Amin, M.A., and Ashour, M.S. (2014). Antimicrobial resistance pattern and their beta-lactamase encoding genes among Pseudomonas aeruginosa strains isolated from cancer patients. *Biomed Res Int* 2014**,** 101635. doi: 10.1155/2014/101635.
