# Supplementary material for: Urban wastewater overflows as hotspots for dissemination of bacteria producing extended-spectrum β-lactamases and carbapenemases in the Suquía River, Argentina
Source: Front Microbiol. 2025 Sep 24;16:1669531. doi: 10.3389/fmicb.2025.1669531 (PMC12504239; doi:10.3389/fmicb.2025.1669531)
Supplement: Supplementary file 8 [file Table_7.docx]

**Table S7. Plasmidic localization prediction of carbapenemase-containing contigs from strains isolated from wastewater overflows (WW) and Suquía River (SR).** The bioinformatic tool Deeplasmid was used to predict the plasmid localization of carbapenemase genetic determinants. The maximum probability of plasmid localization is achieved when the score value is 1. Contig lengths are expressed in base pairs (bp). *N.D.: not determined.

| **Strain (origin)** | **Species** | **Contig length (bp)** | **Deeplasmid**  **score** | **Carbapenemase**  **gene** | **Transferred in**  **mating assays** |
| --- | --- | --- | --- | --- | --- |
| 10Cfr (WW) | *Citrobacter freundii* | 5061 | N.D. | *bla_KPC-2_* | Yes |
| 10Kmi (WW) | *Klebsiella michiganensis* | 18556 | 0.978 | *bla_KPC-2_* | Yes |
| 31Ero (WW) | *Enterobacter roggenkampii* | 24328 | 1.000 | *bla_KPC-2_* | No |
| 34Eho (WW) | *Enterobacter hormaechei* | 116135 | 1.000 | *bla_NDM-1_* | Yes |
| 1.4Eko (SR) | *Enterobacter kobei* | 10561 | 0.935 | *bla_KPC-2_* | No |
| 4.5Aer (SR) | *Aeromonas caviae* | 20725 | 0.999 | *bla_KPC-2_* | No |
